# A framework for transcriptome-wide association studies in breast cancer in diverse study populations

**DOI:** 10.1186/s13059-020-1942-6

**Published:** 2020-02-20

**Authors:** Arjun Bhattacharya, Montserrat García-Closas, Andrew F. Olshan, Charles M. Perou, Melissa A. Troester, Michael I. Love

**Affiliations:** 1 Department of Biostatistics, University of North Carolina-Chapel Hill, Chapel Hill, USA; 2 Division of Cancer Epidemiology and Genetics, National Cancer Institute, Bethesda, USA; 3 Division of Genetics and Epidemiology, Institute of Cancer Research, London, UK; 4 Department of Epidemiology, University of North Carolina-Chapel Hill, Chapel Hill, USA; 5 Lineberger Comprehensive Cancer Center, University of North Carolina-Chapel Hill, Chapel Hill, USA; 6 Department of Genetics, University of North Carolina-Chapel Hill, Chapel Hill, USA; 7 Department of Pathology and Laboratory Medicine, University of North Carolina-Chapel Hill, Chapel Hill, USA

**Keywords:** Transcriptome-wide analysis (TWAS), Breast cancer, Expression quantitative trait loci (eQTL), Survival, Polygenic traits

## Abstract

**Background:**

The relationship between germline genetic variation and breast cancer survival is largely unknown, especially in understudied minority populations who often have poorer survival. Genome-wide association studies (GWAS) have interrogated breast cancer survival but often are underpowered due to subtype heterogeneity and clinical covariates and detect loci in non-coding regions that are difficult to interpret. Transcriptome-wide association studies (TWAS) show increased power in detecting functionally relevant loci by leveraging expression quantitative trait loci (eQTLs) from external reference panels in relevant tissues. However, ancestry- or race-specific reference panels may be needed to draw correct inference in ancestrally diverse cohorts. Such panels for breast cancer are lacking.

**Results:**

We provide a framework for TWAS for breast cancer in diverse populations, using data from the Carolina Breast Cancer Study (CBCS), a population-based cohort that oversampled black women. We perform eQTL analysis for 406 breast cancer-related genes to train race-stratified predictive models of tumor expression from germline genotypes. Using these models, we impute expression in independent data from CBCS and TCGA, accounting for sampling variability in assessing performance. These models are not applicable across race, and their predictive performance varies across tumor subtype. Within CBCS (*N* = 3,828), at a false discovery-adjusted significance of 0.10 and stratifying for race, we identify associations in black women near *AURKA*, *CAPN13*, *PIK3CA*, and *SERPINB5* via TWAS that are underpowered in GWAS.

**Conclusions:**

We show that carefully implemented and thoroughly validated TWAS is an efficient approach for understanding the genetics underpinning breast cancer outcomes in diverse populations.

## Background

Breast cancer remains the most common cancer among women in the world [1]. Breast cancer tends to be more aggressive in young women and African American women, though underlying germline determinants of poor outcomes are not well-studied. Cohorts that represent understudied minority populations, like the Carolina Breast Cancer Study (CBCS), have identified differences in healthcare access, socioeconomics, and environmental exposures associated with disparities in outcome [2–4], but more targeted genomic studies are necessary to interrogate these disparities from a biologic and genetic perspective.

Few genome-wide association studies (GWAS) have studied the relationship between germline variation and survival outcomes in breast cancer, with most focusing instead on genetic predictors of risk [5, 6]. Recently, GWAS have shown evidence of association between candidate common germline variants and breast cancer survival, but these studies are often underpowered [7, 8]. Furthermore, the most significant germline variants identified by GWAS, in either risk or survival, are often located in non-coding regions of the genome, requiring in vitro follow-up experiments and co-localization analyses to interpret functionally [9]. It is important to seek strategies for overcoming these challenges in GWAS, especially because several studies in complex traits and breast cancer risk have shown that regulatory variants not significant in GWAS account for a large proportion of trait heritability [10–12].

Novel methodologic approaches that integrate multiple data types offer advantages in interpretability and statistical efficiency. Escala-García et al. have suggested that aggregating variants by integrating gene expression or other omics may better explain underlying biological mechanisms while increasing the power of association studies beyond GWAS [7]. To alleviate problems with statistical power and interpretability, a recent trend in large-scale association studies is the transcriptome-wide association study (TWAS). TWAS aggregates genomic information into functionally relevant units that map to genes and their expression. This gene-based approach combines the effects of many regulatory variants into a single testing unit that increases study power and provides more interpretable trait-associated genomic loci [13–15]. Hoffman et al. and Wu et al. have recently conducted TWAS for breast cancer risk and have reported several significant associations for genes with breast cancer susceptibility, showing increased power over GWAS [15, 16]. However, these studies either draw from ancestrally homogeneous reference panels like subsets of women of European ancestry from the Genotype-Tissue Expression (GTEx) project [16] or study populations of European descent from the Breast Cancer Association Consortium (BCAC) [15]. It is not known whether these models can be informative in African American women and other groups, though work in race-specific polygenic risk scores suggests that race-specific expression models may be more informative [17]. Recent findings have suggested that stratification by race or ancestry may be necessary to construct proper tests of association across race or ancestry [18, 19]. However, many cohorts, especially large-scale genetic cohorts, may not have a sufficient sample size in minority populations to power these tests.

Here, we provide a framework for TWAS for complex disease outcomes in diverse study populations using transcriptomic reference data from the Carolina Breast Cancer Study (CBCS), a multi-phase cohort that includes an over-representation of African American women [20]. We train race-stratified predictive models of tumor expression from germline variation and carefully validate their performance, accounting for sampling variability and disease heterogeneity, two aspects that previous TWAS in breast cancer have not considered. This framework shows promise for scaling up into larger GWAS cohorts for further detection of risk- or outcome-associated loci (Additional file 4).

## Results

### Race-specific germline eQTL analysis

To assess the association between germline genomic variation and tumor expression of 406 autosomal genes, targeted by the CBCS because of their association with breast cancer progression, we first conducted a full cis-trans expression quantitative trait loci (eQTL) analysis, stratifying on race and controlling for key biological covariates and population stratification (see “Methods”). We discuss the relationship between self-reported race and ancestry in CBCS in Additional file 1: Supplemental Results, showing the relationship between race and genetic ancestry in Additional file 2: Figure S1.

**Fig. 1:**
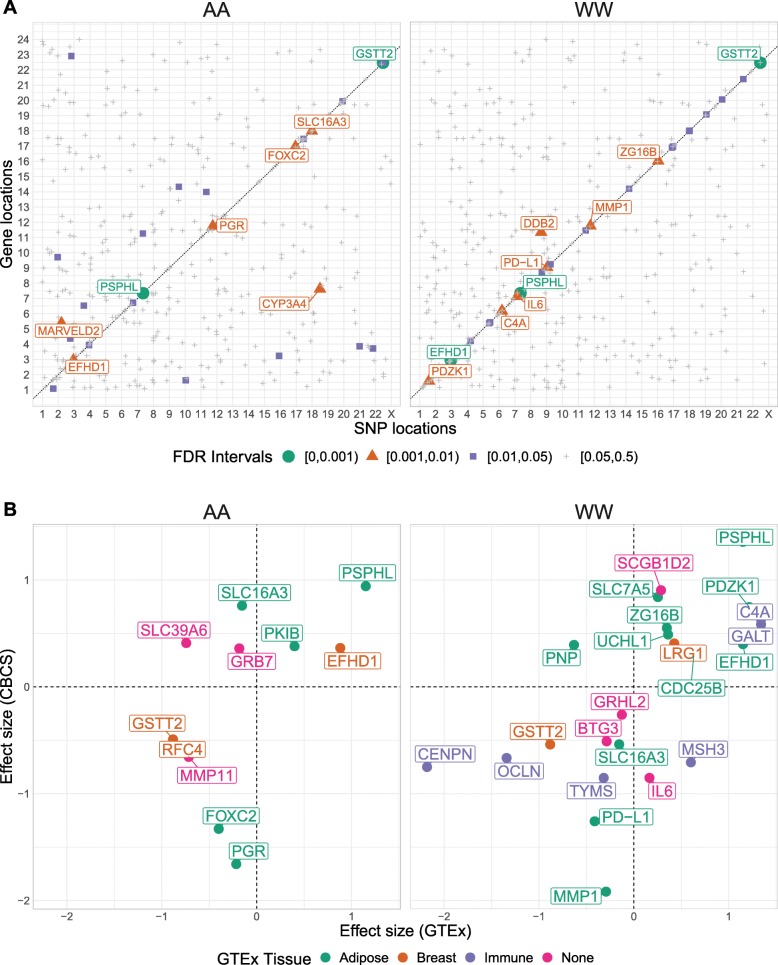
CBCS eQTL results across race and compared with GTEx. **a** Cis-trans plot of top eQTL by gene stratified by self-reported race. Each point represents the top eQTL for a given gene. The color and size of each point reflects the Benjamini-Bogomolov FDR-adjusted *P* value (*BBFDR*) for that eQTL. eGenes with *BBFDR* < 0.01 are labeled. **b** Comparison of effect sizes of eGenes with significant cis-eQTLs in CBCS (*Y*-axis) and GTEx (*X*-axis) over tissue type, stratified by race. eGenes are colored by the GTEx tissue that shows the largest effect size. GTEx effect sizes on the *X*-axis are multiplied by the sign of the correlation between the genotypes of the GTEx and CBCS eSNPs

We evaluated associations between the tumor expression levels of 406 autosomal genes and 5,989,134 germline SNPs in samples derived from 621 self-identified African American women (AA) and 578 self-identified white women (WW). SNPs and genes found in association in an eQTL will be called eSNPs and eGenes, respectively. At a Benjamini-Bogomolov [21] FDR-corrected *P* value (*BBFDR* < 0.05) and after quality control as mentioned in “Methods” (Additional file 2: Figure S2), we identified 266 cis-eQTLs and 71 trans-eQTLs in the AA sample across 32 eGenes, and 691 cis-eQTLs and 15 trans-eQTLs in the WW sample across 24 eGenes. Of these eGenes, 4 are in common across race: *PSPHL*, *GSTT2*, *EFHD1*, and *SLC16A3*. Expression levels of *PSPHL* and *GSTT2* have been previously reported to be governed by respective cis-deletions and serve as distinguishing biomarkers for race [22–25]. The majority of significant eQTLs in both the AA and WW samples were found in cis-association with respective eGenes. However, we saw a higher proportion of significant trans-eQTLs in the AA sample (Additional file 2: Figure S3). The locations and strengths of top eQTLs for all 406 autosomal genes are shown in Fig. 1a, with minor allele frequencies of significant eSNPs plotted in Additional file 2: Figure S4. We followed up this eQTL analysis with a functional enrichment analysis to assess whether significant eQTLs (*BBFDR* < 0.05) overlapped with DNaseI hypersensitive sites in MCF-7 breast cancer cells and/or transcription factor binding sites in T-47D breast cancer cells (see “Methods”). We found that only eQTLs identified in WW women showed significant overlap in both DNaseI cleavage hotspots and transcription factor binding sites in relevant cancer cells at Bonferroni-corrected *P* < 0.05 (Additional file 3: Table S1).

As discussed in detail in Additional file 1: Supplemental Results, we further adjusted our eQTL models for a computationally derived estimate of tumor purity, which showed little effect on the strength and location of top eQTLs by eGene (Additional file 2: Figures S5 and S6). We do not consider tumor purity in any downstream analyses and train predictive models on bulk tumor expression. We also assessed if conditioning on local ancestry would harmonize the eQTL results across race. While 78% of loci had a small increase in significance conditioning on local ancestry, it was not sufficient to bring the tests from the two groups into accordance (Additional file 2: Figure S7). Local ancestry adjustment is discussed further in Additional file 1: Supplemental Results.

We lastly sought to evaluate the source of the significant eQTLs we detect in CBCS. Similarly to previous pan-cancer gerrmline eQTL analyses [26], we cross-referenced eGenes found in CBCS with eGenes detected in relevant healthy tissues from Genotype-Tissue Expression (GTEx) Project: mammary tissue (breast), subcutaneous adipose, and EBV-transformed lymphocytes (immune) (see “Methods”). We attributed all but 7 of the cis-eGenes from CBCS across both AA and WW women found in GTEx to one of these three tissue types (Fig. 1b), with the effect sizes of the top eQTLs for these eGenes correlating very well between CBCS and GTEx (see Additional file 2: Figure S8). We also found adequate overlap of cis-eSNPs in these GTEx tissues and TCGA-BRCA based on the *P* value of SNP-gene association (see Additional file 2: Figure S9). Note that, in GTEx v7, adipose (*N* = 298) has a larger sample size than mammary tissue (*N* = 183) and lymphocytes (*N* = 114). We were unable to replicate CBCS trans-eQTLs in GTEx and TCGA-BRCA [27]. The majority of CBCS trans-eQTLs were identified in AA women, and the sample sizes of individuals of African descent is low in GTEx version 7 and TCGA-BRCA.

### Race-specific predictive models of tumor expression

**Fig. 2:**
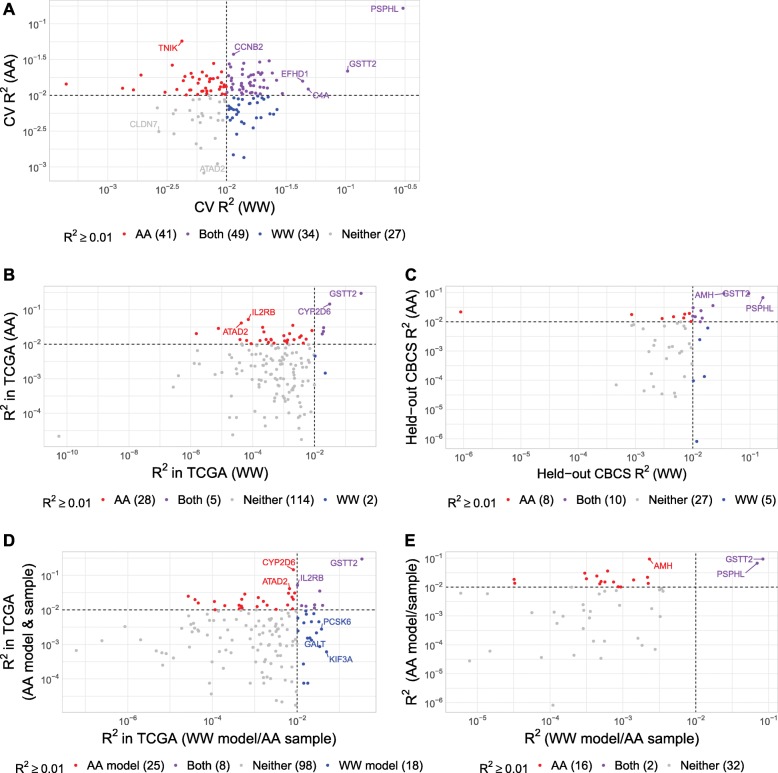
Predictive performance of models in cross-validation, external validation, and across race. **a** Comparison of cross-validation *R*^2^ across race in CBCS. Cross-validation *R*^2^ in CBCS WW women (*X*-axis) and CBCS AA women (*Y*-axis) for each of the 151 analyzed genes. Scales are logarithmic. Dotted lines represent *R*^2^ = 0.01. Colors represent the model with which a given gene can be predicted at *R*^2^ > 0.01. **b** Comparison of validation *R*^2^ across race in TCGA for 149 analyzed genes found in TCGA expression data. **c** Comparison of validation *R*^2^ across race in held-out CBCS samples for 50 analyzed genes. **d** Comparison of *R*^2^ of genes in TCGA AA sample imputed from WW models (*X*-axis) and the AA models (*Y*-axis). **e** Comparison of *R*^2^ of genes in held-out CBCS AA sample imputed from WW models (*X*-axis) and the AA models (*Y*-axis)

Using the significant germline eQTLs of tumor expression as motivation, we used tumor expression and genotyping data from 628 AA women and 571 WW women from CBCS to build predictive models of tumor RNA expression levels for each gene’s breast tumor expression (see “Methods”). Mean cis-heritability (cis-*h*^2^) of the 406 genes is 0.016 (*SE* = 0.019) in AA women and 0.015 (*SE* = 0.019) in WW women, as estimated by GREML-LDMS analysis [28]. For downstream analysis, we only consider genes with cis-*h*^2^ significantly greater than 0 at a nominal *P* value less than 0.10 from the relevant likelihood ratio test. Considering only these genes, the mean cis-*h*^2^ of genes is 0.049 (*SE* = 0.016) in AA models and 0.052 (*SE* = 0.016) in WW models. Of the predictive models built for these genes, 125 showed a fivefold cross-validation prediction performance (CV *R*^2^) of at least 0.01 (10% Pearson correlation between predicted and observed expression with *P* < 0.05) in one of the two predictive models. Figure 2a shows the CV *R*^2^ of these 153 genes across race. The median CV *R*^2^ for the 153 genes was 0.011 in both AA and WW women. Cis-*h*^2^ and CV *R*^2^ are compared in Additional file 2: Figure S10. We also show mean CV and external validation (EV) *R*^2^ with quantiles for prioritized genes across the training set and both external test sets in Additional file 3: Table S2.

Based on model performance in CBCS, we selected 46 genes in AA women and 57 genes in WW women for association analyses between predicted tumor gene expression and breast cancer survival, using data from all patients from CBCS with genotype data. These genes were selected because they showed a CV *R*^2^ > 0.01 (10% correlation between observed and predicted expression in the CBCS training set) and cis-*h*^2^ ≥ 0 with nominal *P* < 0.10 in a given race strata.

### Evaluation of predictive models in independent data

Predictive performance was strong across race and biological and molecular subtype in two external samples: The Cancer Genome Atlas (TCGA) and a held-out CBCS sample set. We defined the imputed expression of a given gene in an external cohort as the GReX, or the germline genetically regulated tumor expression, of that gene.

The first sample is derived from TCGA breast tumor tissues with 179 AA and 735 WW women. We compared predictive performance by calculating an external validation *R*^2^ (EV *R*^2^) with squared Spearman correlations. Of the 151 genes modeled in CBCS training data with significant cis-*h*^2^, 149 genes were measured via RNA-seq in TCGA. A comparison of predictive performance in TCGA for these 149 genes is shown in Fig. 2b, showing adequate performance in AA women (33 genes with EV *R*^2^ > 0.01) and poor performance in WW women (7 genes with EV *R*^2^ > 0.01). The top predicted gene in cross-validation from CBCS for both races, *PSPHL*, was not present in the TCGA normalized expression data and could not be validated. Another top cross-validated gene, *GSTT2*, was present in TCGA expression data and was validated as the top genetically predicted gene in TCGA by EV *R*^2^.

We also imputed expression into entirely held-out samples from CBCS data (1121 AA and 1070 WW women) that have gene expression for a subset of the genes (166 of 417 genes) in the CBCS training set. These samples were largely derived from Phases I and II of CBCS (see “Methods”). A comparison of imputation performance in CBCS for 50 genes (genes with cis- *h*^2^ ≥ 0.01 in CBCS training set) is shown in Fig. 2c, showing adequate performance in both AA and WW women (18 and 15 genes with EV *R*^2^ > 0.01 in AA and WW women).

### Predictive models are not applicable across race

We find that the predictive accuracy of most genes was lower when expression was imputed in AA women using models trained in the WW sample. We employed the WW predictive models to impute expression into AA samples from TCGA and held-out CBCS data. We compare the performances of the WW model and AA model in the AA sample in Fig. 2d (TCGA) and 2e (CBCS). In held-out CBCS samples, with the WW model, we could only predict *PSPHL* and *GSTT2* at *R*^2^ > 0.01 in the AA sample, as the expression of these genes is modulated mostly by strongly associated cis-eSNPs. In TCGA, our WW models performed adequately in AA women, though the WW models predicted fewer genes at *R*^2^ > 0.01 than the AA models.

### Evaluation of predictive performance across subtype

While predictive accuracy of expression models was stable across datasets, there was greater heterogeneity across biological and molecular subtype. In part, this is due to small sample sizes within race and subtype-specific strata. Upon first inspection, we see vast differences in the performance of our models across subtype (Additional file 2: Figure S11), with a large majority of genes performing at EV *R*^2^ > 0.01 in rarer subtypes, like HER2-enriched breast cancers. However, we recognized sample sizes in the TCGA validation set were relatively small, especially when considering AA women and women of certain subtype, e.g., as low as 16 AA women with HER2-enriched breast cancer. As overall correlation between observed and imputed expressions are near 0, we sought to account for sampling variability when imputing into groups of women with such small sample sizes.

**Fig. 3:**
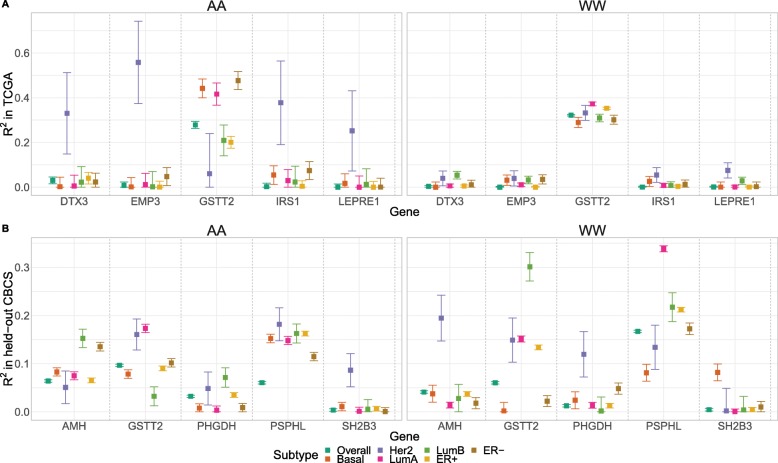
Predictive performance of key genes, accounting for sampling variability*.* Validation *R*^2^ across PAM50 molecular subtype and estrogen receptor status, stratified by race, for example genes with highly variable *R*^2^ in TCGA (**a**) and held-out CBCS (**b**). Squared Spearman correlation (*Y*-axis), denoted *R*^2^, between observed and predicted gene expression is plotted for different genes (*X*-axis), stratified by PAM50 subtype and estrogen receptor status. Points are colored and shaped according to subtype. Error bars provide 90% confidence intervals inverted from the corresponding permutation test

We employed a permutation scheme: permuting observed expression values among samples 10,000 times to generate a null distribution for EV *R*^2^. We then tested for the null hypothesis *R*^2^ = 0, controlling for false discovery, according to this null distribution. Additional file 2: Figure S12 displays *q*-values in Manhattan form [29], showing that the proportion of genes with EV *R*^2^ significantly different from 0 is similar across subtypes. We inverted this permutation test [30] to construct a confidence interval for EV *R*^2^. We find that the EV *R*^2^ of several genes are highly variable across subtypes, even when accounting for differences in sample size and therefore sampling variation. Key examples of such genes with variable EV *R*^2^ across subtypes are shown in Fig. 3. We also find little effect of GReX on PAM50 subtype calls (Additional file 2: Figure S13), with more details in Additional file 1: Supplemental Results.

### Predicted expression associated with breast cancer-specific survival

**Fig. 4:**
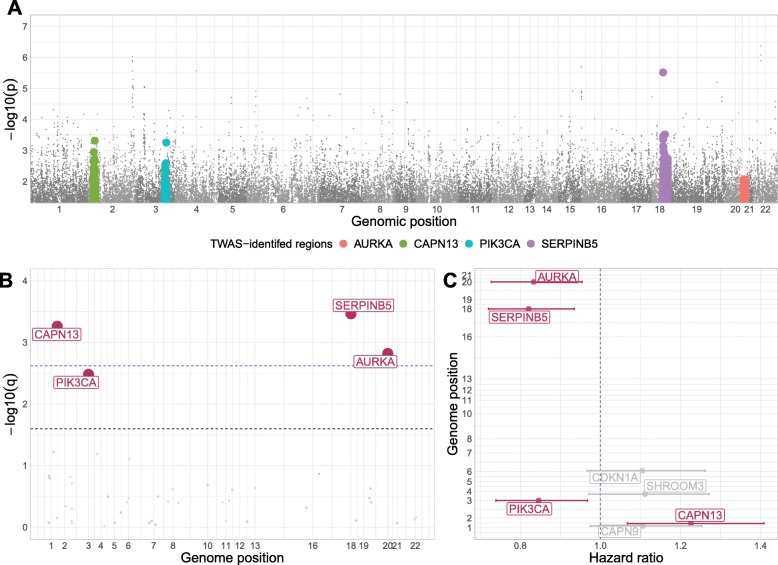
GWAS and TWAS results in AA women. **a** Manhattan plot of traditional GWAS on breast cancer survival. Genomic regions found to be significantly associated with survival in TWAS are represented in various colors. No SNVs reach Benjamini-Hochberg FDR-adjusted genome-wide significance. **b** Manhattan plot of TWAS on breast cancer survival. Genomic regions found to be significant at FDR-adjusted *P* < 0.10 are highlighted in red. The blue line represents a cutoff of FDR-adjusted *α* = 0.05 and the dotted black line represents a cutoff of FDR-adjusted *α* = 0.10. **c** Caterpillar plot of log-hazard rates with FDR-adjusted 90% confidence levels (*X*-axis) and genomic position (*Y*-axis). Results shown are significant at nominal *P* < 0.10. Genes highlighted in red represent genes with GReX significantly associated with survival at FDR-adjusted *P* < 0.10

To assess association between imputed gene expression and breast cancer-specific survival, we constructed race-stratified cause-specific proportional hazard models for 3828 samples from CBCS (1865 AA and 1963 WW), where we model time to mortality due to breast cancer. We find high power of detection of survival-associated loci over genes with varied cis-heritabilities (Additional file 2: Figure S16), with details included in Additional file 1: Supplemental Results. Of the genes evaluated, we detected 4 whose GReX were associated with breast cancer-specific survival at FDR-adjusted *P* < 0.10 in AA women, shown in Table 1 and Fig. 4. We did not identify any genes with GReX associated with survival in WW women.

**Table 1:** Genes with GReX found in association with breast cancer-specific survival in AA women

Region	Gene	Hazard ratio (90% CI)^a^	*Z*-statistic^a^	*P* value^a^	GReX *R*^2^ (*h*^2^)^b^
*20q13.2*	*AURKA*	0.83 (0.73, 0.95)	−2.52	1.5 × 10^−3^	0.021 (0.055)
*2p23.1*	*CAPN13*	1.22 (1.07, 1.41)	2.76	5.4 × 10^−4^	0.011 (0.047)
*3q26.32*	*PIK3CA*	0.85 (0.74, 0.97)	−2.34	3.2 × 10^−3^	0.020 (0.033)
*18q21.33*	*SERPINB5*	0.82 (0.72, 0.93)	−2.85	3.4 × 10^−4^	0.010 (0.026)

^a^Hazard ratio and FDR-adjusted 90% confidence intervals, *Z*-statistic, and *P* value of association of GReX with breast cancer-specific survival

^b^Cross-validation *R*^2^ of gene expression in AA models

An association between increased GReX and increased risk of breast cancer-specific mortality was identified for *CAPN13* (*2p23.1*). We also found protective associations between higher GReX of *AURKA (20q13.2)*, *PIK3CA (3q26.32)*, and *SERPINB5* (*18q21.33*) and lower risk of breast cancer mortality (Fig. 4c). Of these 4 loci, associations with survival have been reported with SNPs near the same chromosomal region as *AURKA*, *PIK3CA,* and *SERPINB5* [8, 31–35], though none of these reported SNPs were utilized in constructing the GReX of this gene. Furthermore, the GReX of these four genes were not significantly correlated (*P* > 0.05 for all pairwise Spearman correlation tests), and the sets of SNPs used in constructing the GReX of these four genes had no pairwise intersections, providing evidence that their independent association with breast cancer-specific survival was not a pleiotropic effect from shared or correlated SNPs.

To determine whether the associations between predicted gene expression and breast cancer-specific survival were independent of GWAS-identified association signals, we performed conditional analyses adjusted for the most significant GWAS-identified survival-associated SNPs closest to the TWAS-identified gene by adjusting the cause-specific proportional hazards model for the genotype from this SNP. We found that the association for *PIK3CA* had a small change in effect size after adjustment for its adjacent survival-associated SNP, and its SNP-adjusted association was insignificant, while the other genes’ associations remained significant after adjustment (Table 2). This conditional analysis suggests that the GReX of *AURKA*, *CAPN13*, and *SERPINB5* may be associated with breast cancer-specific survival independent of the GWAS-identified variant. No previously reported survival-associated SNPs were found significant at the genome-wide significance level in our dataset, and none of the closest survival-associated SNPs used in conditional adjustment were significant (Fig. 4a). This supports our observation that correctly analyzed TWAS using relevant tissue gene expression may increase power for association testing.

**Table 2:** Genes with GReX found in association with breast cancer-specific survival

Gene	Closest survival-associated SNP^a^	Distance to closest survival-associated SNP^a^	Hazard ratio, adjusting for adjacent GWAS-SNP (90% CI)^b^	*P* value, adjusting for adjacent risk SNPs^b^
*AURKA*	rs202100873	87.1 kb	0.84 (0.74, 0.94)	0.027
*CAPN13*	rs72068647	266.9 kb	1.18 (1.04, 1.33)	0.046
*PIK3CA*	rs66487567	271.9 kb	0.88 (0.78, 1.00)	0.096
*SERPINB5*	rs376302305	89.4 kb	0.84 (0.75, 0.94)	0.028

^a^Top survival-associated SNP in cis-region of the given gene from GWAS for survival and distance of top cis-SNP from gene

^b^FDR-adjusted hazard ratio, 90% confidence interval, and *P* value for association of GReX and breast cancer-specific survival, adjusting for adjacent survival-associated SNPs

As we deal with case-only data, we wished to inspect any collider bias that arises from unmeasured confounders that are associated with both breast cancer incidence and survival (see Additional file 2: Figure S17) [36]. Since a case-control dataset was not readily available to us to test associations between the GReX of genes with breast cancer risk, we construct the weighted burden test, as in FUSION [14], for the GReX of *AURKA*, *CAPN13, PIK3CA*, and *SERPINB5* in the GWAS summary statistics for breast cancer risk in AA women available from BCAC using the iCOGs dataset and additional GWAS [37–39]. We find that none of the GReX of these genes are significantly associated with breast cancer incidence (*Z* > 1.96, *P* < 0.05), suggesting minimal presence of collider bias in our estimates of association with survival for the GReX of these four genes.

Lastly, we examined the association of the GReX of these four genes with breast cancer-specific survival in AA women, stratified by estrogen receptor (ER) subtype. We find that overall associations with survival are often driven by significant associations in a single subtype, though there is evidence of significant hazardous association in both ER subtypes for *CAPN13* (Additional file 2: Figure S14). We also did not detect a survival association with the total expression of these 4 genes, as estimated from breast cancer-specific Cox models (Additional file 2: Figure S15).

## Discussion

In this paper, we studied the relationship between breast cancer-specific survival and germline genetics using a TWAS framework. This study is the first systematic TWAS for breast cancer-specific survival, motivated by a full cis-trans eQTL analysis with one of the largest sample sizes for breast tumor gene expression in African American women. Our analyses underscore the importance of accounting for sampling variability when validating predictive models for TWAS and incorporating race or ancestry in these models, an aspect which confounds naïve comparisons involving imputed GReX across validation subgroups of different sample size.

Our race-stratified eQTL analysis reveals a strong cis-signal between germline variants and tumor expression of several genes, which is both differential across race and not exclusively attributable to healthy breast tissue. We also identified considerably more trans-eQTLs in the AA sample. This result may reinforce race differences in eQTL architecture as the ratio of detected trans-eQTLs to cis-eQTLs is not directly linked to sample size [40]. Differences in allele frequencies and linkage disequilibrium may contribute to observed differences in cis-eQTLs, as reported by Mogil et al. [18], and we hypothesize that such differences may likewise affect trans-eQTLs. Alternatively, there is a prevailing thought in literature about trans genetic regulation in admixed populations that the genetic diversity in individuals of African ancestry leads to added power of eQTL detection [41, 42]. These race differences in eQTLs motivated the racial stratification of our predictive expression models [43]. We discuss both in-sample and out-of-sample predictive performance in Additional file 1: Supplemental Results.

An important implication of our work is the race-specificity of TWAS methods. We find that expression models trained in WW women generally have poor performance in AA women. Epidemiological studies have stressed accounting for differences in race by stratification or adjustment for admixture estimates when constructing polygenic scores [44]. Our observations suggest that this epidemiological note of caution extends to creating predictive models for RNA expression. Previous TWAS studies of breast cancer risk have either used models trained in a sample of predominantly European ancestries [16] or imputed into large cohorts of strictly patients of European descent [15]. Hoffman et al. exclude SNPs that were monomorphic in any of the 14 different ancestral populations they analyze [16], though this may not capture all effects of ancestry on genetic regulation of expression, including the possibility for interactions. We contend that accounting for ancestry or stratifying by race may be necessary to draw correct inference in large, ancestrally heterogeneous cohorts.

Our data also suggests that predictive performance may vary by molecular subtype. Previous groups have shown the predictive utility of catering polygenic risk scores to breast cancer subtype [45, 46], a phenomenon we investigated in our predictive models of tumor expression. Even after accounting for sampling variability in prediction, we found that several genes have varied degrees of GReX across subtype and race. Not only does this finding suggest that TWAS predictive models may need to account for subtype heterogeneity, we reinforce the importance of sampling variability in validation of predictive models in external cohorts. For example, Wu et al. trained their models in a relatively small set of 67 women from GTEx and validated their 12,824 models in a validation set of 86 women from TCGA without accounting for sampling variability of predictive performance [15]. A recent multi-tissue TWAS in ovarian cancer from Gusev et al. considered validation of their predictive models by leveraging multiple independent cohorts to assess replication rates [47]. We recommend such an approach if multiple independent cohorts are accessible. But, in TWAS evaluation in a single tissue, studies should place a strong emphasis on validation, accounting for sampling variability of prediction *R*^2^ prior to imputation in larger cohorts.

While many of the most significant findings here are methodological in nature, we also have data to suggest that four genomic loci in AA women may merit further investigation relative to breast cancer survival. Two of these 4 TWAS-identified genes have strong functional evidence in breast cancer survival literature. Mutations in *AURKA* and *PIK3CA* have previously been shown to be significantly associated with breast cancer survival rates [31–33]. Less is known about the involvement of *SERPINB5* and *CAPN13* in breast cancer survival, though they have been identified in studies into breast cancer progression [48–52]. These four loci merit further studies for validation and functional characterization, both in large GWAS cohorts and using in vitro studies. We did not observe any significant association between the total expression of these 4 genes and breast cancer-specific survival. This suggests that the germline-regulated component of the tumor expression of these genes—a small fraction of the total expression variation—may be associated with survival outcomes. Numerous factors, including copy number alterations, epigenetic or post-transcriptional regulation, and exposures and technical artifacts in measurement contributed to the total expression measured in the tumor. Thus, we do not expect that significant GReX association implies total expression association, or vice versa.

We also observed that 3 of the 4 associations were driven by very strong effect sizes within a single subtype. Though we cannot contextualize this result, it highlights an often-overlooked modeling consideration. In a cohort that is both biologically and ancestrally heterogeneous, as in CBCS, investigators should consider modeling choices beyond simple linear adjustments for subtype and race. Akin to the logic of Begg et al. and Martínez et al., it may be prudent in future TWAS to stratify predictive models on both race and biological subtype to increase power to detect outcome-associated loci that are strongly present within only one such strata or have heterogeneous effects across strata [53, 54].

Since the CBCS analysis was a case-only study, we were wary of potential collider bias by unmeasured confounders associated with both breast cancer risk and progression [36, 55–57], which may affect the effect sizes of association between survival and GReX of genes. None of the GReX of these four genes showed significant transcriptome-wide associations with breast cancer risk in iCOGs data [37–39], suggesting that our estimates of association may be free of the collider bias. As Escala-García et al. highlights, germline variation can affect breast cancer prognosis via tumor etiology (risk of developing a tumor of a certain subtype), or via mechanisms that are relevant post-tumorigenesis, such as the cellular response to therapy or the host-tumor micro-environment [7]. Ideally, in future TWAS and integrated omic analyses of breast cancer survival, it is prudent to consider joint models of breast cancer risk and survival to account for pleiotropic effects of germline genotype and any associations with unmeasurable confounders [56].

One limitation of our study is that data on somatic amplifications and deletions were not yet available for the CBCS cohort we analyzed. Removing the somatic copy number variation signal from tumor expression profiles may improve our estimates of cis-heritability and perhaps the predictive performance of our models, though previous TWAS in ovarian cancer shows the effect to be qualitatively small (approximately less than 2% change in heritability) [47]. Furthermore, not all genes in the CBCS Nanostring panel have a significant heritable component in expression regulation. These genes, like *ESR1*, which have a significant role in breast cancer etiology [58], could not be investigated in our study. Lastly, since CBCS mRNA expression is assayed by the Nanostring nCounter system, we could only analyze 94 aggregated locations on the human transcriptome across race. However, the Nanostring platform allows the CBCS to robustly measure expression from FFPE samples on a targeted panel of breast cancer and race-related genes, allowing us to leverage the large sample size from all three phases of the CBCS. One of the greatest strengths of our study is that the CBCS affords us both a large training and test set of AA and WW women for race-stratified predictive models. Such data is important in drawing inference in more ancestrally heterogeneous populations. Accordingly, the statistical power of our study is high to detect associations for genes with relatively high cis-heritability. Future studies in large GWAS cohorts, such as those within the Breast Cancer Association Consortium, will elucidate how to account for ancestral and biological heterogeneity in detecting survival-associated loci.

## Conclusion

We have provided a framework of transcriptome-wide association studies (TWAS) for breast cancer outcomes in diverse study populations, considering both ancestral and subtype-dependent biological heterogeneity in our predictive models. From a more theoretical perspective, this work will inform the utilization of TWAS methods in polygenic traits and diverse study populations, stressing rigorous validation of predictive models prior to imputation and careful modeling to capture associations with outcomes of interest in diverse populations.

## Methods

### Data collection

#### Study population

The Carolina Breast Cancer Study (CBCS) is a population-based study conducted in North Carolina (NC) that began in 1993; study details and sampling schemes have been described in previous CBCS work [20, 59]. Patients of breast cancer aged between 20 and 74 years were identified using rapid case ascertainment in cooperation with the NC Central Cancer Registry, with self-identified African American and young women (ages 20–49) oversampled using randomized recruitment [20]. Randomized recruitment allows sample weighting to make inferences about the frequency of subtype in the NC source population. Details regarding patient recruitment and clinical data collections are described in Troester et al. [2].

Date of death and cause of death were identified by linkage to the National Death Index. All diagnosed with breast cancer have been followed for vital status from diagnosis until date of death or date of last contact. Breast cancer-related deaths were classified as those that listed breast cancer (International Statistical Classification of Disease codes 174.9 and C-50.9) as the underlying cause of death on the death certificate. By the end of follow-up, we identified 674 deaths, 348 of which were due to breast cancer. In total, we compiled 3828 samples (1865 AA and 1963 WW) from all phases of CBCS with relevant survival and clinical variables. All 3828 samples have associated germline genotype data. Of these 3828 samples, we consider 1388 (621 AA and 578 WW) samples with Nanostring nCounter expression data for eQTL analysis and training of predictive expression models.

#### CBCS genotype data

Approximately 50% of the SNPs for the OncoArray were selected as a “GWAS backbone” (Illumina HumanCore), which aimed to provide high coverage for the majority of common variants through imputation. The remaining SNPs were selected from lists supplied by six disease-based consortia, together with a seventh list of SNPs of interest to multiple disease-focused groups. Approximately 72,000 SNPs were selected specifically for their relevance to breast cancer. The sources for the SNPs included in this backbone, as well as backbone manufacturing, calling, and quality control, are discussed in depth by the OncoArray Consortium [60]. All samples were imputed using the October 2014 (v.3) release of the 1000 Genomes Project dataset [61] as a reference panel in the standard two-stage imputation approach, using *SHAPEIT2* for phasing and *IMPUTEv2* for imputation [62–64]. All genotyping, genotype calling, quality control, and imputation was done at the DCEG Cancer Genomics Research Laboratory [60].

From the provided genotype data, we excluded variants (1) with a minor frequency less than 1% based on genotype dosage and (2) that deviated significantly from Hardy-Weinberg equilibrium at *P* < 10^−8^ using the appropriate functions in *PLINK v1.90b3* [65, 66]. Finally, we intersected genotyping panels for the AA and WW samples, resulting in 5,989,134 autosomal variants and 334,391 variants of the X chromosome. CBCS genotype data was coded as dosages, with reference and alternative allele coding as in the National Center for Biotechnology Information’s Single Nucleotide Polymorphism Database (dbSNP).

#### CBCS gene expression data

Paraffin-embedded tumor blocks were requested from participating pathology laboratories for each sample, reviewed, and assayed for gene expression using Nanostring as discussed previously [2]. In total, 1388 samples with invasive breast cancer from the CBCS were analyzed for a total of 406 autosomal genes and 11 genes on the X chromosome. All assays were performed in the Translational Genomics Laboratory at the University of North Carolina at Chapel Hill.

We used the *NanoStringQCPro* package in Bioconductor to first eliminate samples that did not have sufficient Nanostring data quality [67]. Next, we normalized distributional differences between lanes with upper-quartile normalization [68]. Unwanted technical and biological variation (i.e., tissue heterogeneity) was estimated in the resulting gene expression data with techniques from the *RUVSeq* package from Bioconductor [69]. Unwanted variation was controlled using the distribution of 11 endogenous housekeeping genes on the Nanostring gene expression panel. Ultimately, we removed two dimensions of unwanted variation from the variance-stabilized transformation of the gene expression data [70, 71]. We lastly used principal component analysis to detect and remove any significant, potential outliers. A final intersection of samples that had both genotype and gene expression data gave us a final sample of 1199 subjects (628 AA women and 571 WW women).

#### TCGA genotype data

Birdseed genotype files of 914 of WW and AA women were downloaded from the Genome Data Commons (GDC) legacy (GRCh37/hg19) archive. Genotype files were merged into a single binary PLINK file format (BED/FAM/BIM) and imputed using the October 2014 (v.3) release of the 1000 Genomes Project dataset as a reference panel in the standard two-stage imputation approach, using SHAPEIT v2.837 for phasing and IMPUTE v2.3.2 for imputation [62–64]. We excluded variants (1) with a minor allele frequency of less than 1% based on genotype dosage, (2) that deviated significantly from Hardy-Weinberg equilibrium (*P* < 10^−8^) using appropriate functions in PLINK v1.90b3 [65, 66], and (3) located on sex chromosomes. We further excluded any SNPs not found on the final, quality-controlled CBCS genotype data. Final TCGA genotype data was coded as dosages, with reference and alternative allele coding as in dbSNP.

#### TCGA expression data

TCGA level-3 normalized RNA expression data were downloaded from the Broad Institute’s GDAC Firehose (2016/1/28 analysis archive) and subsetted to the 417 genes analyzed in CBCS. A total of 412 of these 417 were available in TCGA expression data.

### Computational methods

#### Deconvolution of bulk tumor RNA

A study pathologist analyzed tumor microarrays (TMAs) from 176 of the 1199 subjects to estimate area of dissections originating from epithelial tumor, assumed here as a proxy for the proportion of the bulk RNA expression attributed to the tumor. Using these 176 observations as a training set and the normalized gene expressions as the design matrix, we trained a support vector machine model tuned over a 10-fold cross-validation [72, 73]. The cross-validated model was then used to estimate tumor purities for the remaining 1023 samples from their gene expressions. We do not consider tumor purity in final eQTL models and all downstream analyses.

#### eQTL analysis

Using the 1199 samples (621 AA, 578 AA) with expression data, we assessed the additive relationship between the gene expression values and genotypes with linear regression analysis using *MatrixeQTL* [74], in the following model:

$$ {E}_g={X}_s{\beta}_s+{X}_C{\beta}_C+{\epsilon}_g, $$

where *E*_*g*_ is the gene expression of gene *g*, *X*_*s*_ is the vector of genotype dosages for a given SNP *s*, *C* is a matrix of covariates, *β*_*s*_ and *β*_*C*_ are the effect sizes on gene expression for the SNP *s* and the covariates *C*, respectively, and *ϵ* is assumed to be Gaussian random error with mean 0 and common variance *σ*^2^ for all genes *g*.

We calculated both cis- (variant-gene distance less than 500 kb) and trans-associations between variants and genes. Classical *P* values were calculated for Wald-type tests of *H*_0_ : *β*_*s*_ = 0 and were adjusted post hoc via the Benjamini-Bogomolov hierarchical error control procedure, *TreeQTL* [21]. We conducted all eQTL analyses stratified by race. Age, BMI, postmenopausal status, and the first 5 principal components of the joint AA and WW genotype matrix were included in the models as covariates in *C*. Estimated tumor purity was also included as a covariate to assess its impact on strength and location of eQTLs. Any SNP found in an eQTL with Benajmini-Bogomolov adjust *P* value *BBFDR* < 0.05 is defined as an eSNP using *TreeQTL* [21]. The corresponding gene in that eQTL is defined as an eGene. We exclude samples with Normal-like subtype, as classified by the PAM50 classifier, due to generally low tumor content. We developed a formal quality control procedure to follow-up on significant eQTLs by defining further MAF cutoff based on additive genotypes (i.e., 0,1, and 2 copies of the minor allele) and rigorous visual inspection (i.e., Additional file 2: Figure S2).

We downloaded healthy tissue eQTLs from the Genotype-Tissue Expression (GTEx) Project and cross-referenced eGenes and corresponding eSNPs between CBCS and GTEx in healthy breast mammary tissue, EBV-transformed lymphocytes, and subcutaneous adipose tissue. We considered these tissues mainly due to their high relative composition in bulk breast tumor samples, as shown previously in many studies [75–78]. The Genotype-Tissue Expression (GTEx) Project was supported by the Common Fund of the Office of the Director of the National Institutes of Health, and by NCI, NHGRI, NHLBI, NIDA, NIMH, and NINDS. The data used for the analyses described in this manuscript were obtained from the GTEx Portal on 05/12/19.

#### Functional enrichment of eQTLs

We assessed whether significant eQTLs (*BBFDR < 0.05*) were functionally enriched in DNaseI cleavage hotspots in the MCF-7 breast cancer cell line, ESR1 transcription factor (TF) binding sites in the T-47D breast cancer cell line, and any TF binding sites in the T-47D breast cancer cell line, downloaded from the ENCODE consortium repository [79, 80]. Data for DNaseI hypersensitive sites were generated by the UW ENCODE group [81, 82]. ChIP-seq data used in the TF binding site analysis was generated by the Myers Lab at the HudsonAlpha Institute for Biotechnology and by the labs of Michael Snyder, Mark Gerstein, Sherman Weissman at Yale University, Peggy Farnham at the University of Southern California, Kevin Struhl at Harvard, Kevin White at the University of Chicago, and Vishy Iyer at the University of Texas, Austin. These data were processed into uniform peak calls by the ENCODE Analysis Working Group pipeline developed by Anshul Kundaje. The clustering of the uniform peaks was performed by UCSC. The Factorbook motif identifications and localizations (and valuable assistance with interpretation) were provided by Jie Wang, Bong Hyun Kim, and Jiali Zhuang of the Zlab (Weng Lab) at UMass Medical School [83–85].

eQTL functional enrichment was categorized using *QTLtools* [86] to count the observed number of eQTLs found in a 1-kb window of a functional annotation and estimate the mean expected number of eQTLs found near the annotation over 10,000 replications. Fisher’s exact test was then used to estimate the odds ratio, 95% confidence interval, and *P* value to assess how the observed number of eQTLs and the mean expected number of eQTLs differ, as described by Delaneau et al. [86].

#### Local ancestry adjustment for cis-eQTLs

For cis-eGenes that were identified in only one of AA or WW women, we followed up with a cis-eQTL analysis adjusted for inferred local ancestry. Reference genotypes were downloaded from the 1000 Genomes Project version 3 for Utah residents with Northern and Western European ancestry (CEU) and Yoruban individuals from Ibadan, Nigeria (YRI) [61]. Phased genotypes from the assumed admixed samples from CBCS were then compared to reference genotypes using RFMix v1.5.4 to estimate the posterior probability of CEU and YRI ancestry at a given haplotype, which is converted to an estimated dosage of inherited YRI alleles [87, 88]. We then follow Zhong et al.’s framework for adjusting eQTLs by estimated local ancestry [89]. Briefly, for gene expression *g*, dosage of SNP of interest *s*, covariates *X*_*C*_, and estimated local ancestry *l* for the given SNP, we first residualize and scale to zero mean and unit variance *g*, *s*, and *l* by *X*_*C*_. We then fit the following linear model to estimate the local ancestry-adjusted eQTL effects:

$$ \overset{\sim }{g}=\overset{\sim }{s}+\overset{\sim }{l}+\epsilon, $$

where $\overset{\sim }{g},\overset{\sim }{s},$ and $\overset{\sim }{l}$ are the residualized and scaled gene expression, SNP dosage, and estimated local ancestry, respectively [89].

#### Estimation of cis-heritability

Cis-heritability (cis-*h*^2^) using genotypes within 500 kb of the gene of interest was estimated using the GREML-LDMS method, proposed to estimate heritability by correction for bias in linkage disequilibrium (LD) in estimated SNP-based heritability [28]. We do not consider the trans components in heritability estimation. Analysis was conducted using *GCTA* v.1.92 [90]. Briefly, Yang et al. shows that estimates of heritability are often biased if causal variants have a different minor allele frequency (MAF) spectrums or LD structures from variants used in analysis. They proposed an LD and MAF-stratified GREML analysis, where variants are stratified into groups by MAF and LD, and genetic relationship matrices (GRMs) from these variants in each group are jointly fit in a multi-component GREML analysis. Extensive details are given by Yang et al. [28].

For downstream analysis, we only consider the 151 genes (81 in AA women and 100 in WW women) with cis- *h*^2^ that can be estimated with nominal *P* value <0.10.

#### Predictive tumor expression models

We adopt general techniques from PrediXcan and FUSION to estimate eQTL-effect sizes for predictive models of tumor expression from germline variants [13, 14]. First, gene expressions were residualized for the covariates *C* included in the eQTL models (age, BMI, postmenopausal status, and genotype PCs) given the following ordinary least squares model:

$$ {E}_g={X}_C{\beta}_C+{\epsilon}_g. $$

We then consider downstream analysis on ${\overset{\sim }{E}}_g\equiv {E}_g-{X}_C{\hat{\beta}}_C$.

For a given gene *g*, we consider the following linear predictive model:

$$ {\overset{\sim }{E}}_g={X}_g{w}_g+{\epsilon}_g, $$

where ${\overset{\sim }{E}}_g$ is the gene expression of gene *g*, residualized for the covariate matrix *X*_*C*_, *X*_*g*_ is the genotype matrix for gene *g* that includes all cis-SNPs for gene *g* (within 500 kb of either the 5′ or 3′ end of the gene) and all trans-eQTLs with *BBFDR* < 0.01, *w*_*g*_ is a vector of effect sizes for eQTLs in *X*_*g*_, and *ϵ*_*g*_ is Gaussian random error with mean 0 and common variance for all *g*.

We estimate *w*_*g*_ with the best predictive of three schemes: (1) elastic-net regularized regression with mixing parameter *α* = 0.5 and *λ* penalty parameter tuned over fivefold cross-validation [13, 91], (2) linear mixed modeling where the genotype matrix *X*_*g*_ is treated as a matrix of random effects and ${\hat{w}}_g$ is taken as the best linear unbiased predictor (BLUP) of *w*_*g*_, using *rrBLUP* [92], and (3) multivariate linear mixed modeling as described above, estimated using *GEMMA* v.0.97 [93].

In these models, the genotype matrix *X*_*g*_ is pruned for linkage disequilibrium (LD) prior to modeling using a window size of 50, step size of 5, and LD threshold of 0.5 using *PLINK* v.1.90b3 [66] to account for redundancy in signal. We believe that our LD-pruning thresholds and window sizes are not stringent [94] and noticed that LD-pruning the design matrix of genotypes lead to greater CV *R*^2^ (Additional file 2: Figure S18). The final vectors ${\hat{w}}_g$ of effect sizes for each gene *g* are estimated by the estimation scheme with the best fivefold cross-validation performance. All predicted models are stratified by race, i.e., an individual model of tumor expression for AA women and WW women for each gene *g*.

To impute expression into external cohorts, we then construct the germline genetically regulated tumor expression *GReX*_*g*_ of gene *g* given ${\hat{w}}_g$ in the predictive model as follows:

$$ GRe{X}_g={X}_{g, new}{\hat{w}}_g, $$

where *X*_*g*, *new*_ is the genotype matrix of all available SNPs in the feature set of ${\hat{w}}_g$ in a GWAS cohort.

All final models are available here: https://github.com/bhattacharya-a-bt/CBCS_TWAS_Paper.

#### Validation in TCGA

Using our stratified predictive models of tumor expression, we imputed expression in TCGA and measured predictive accuracy of each gene through prediction *R*^2^, defined here as the squared Spearman correlation between observed and imputed expression. It is important to note that all variants in the CBCS-trained predictive models are not represented in the TCGA genotype data. Predictive performance in TCGA was also assessed stratified by PAM50 intrinsic subtype and estrogen receptor status.

To account for sampling variability in calculating correlations in validation cohorts of smaller sample sizes, we calculated a permutation null distribution for each gene by permuting observed expressions 10,000 times and calculating a “null” prediction *R*^2^ at each permutation. The sample validation prediction *R*^2^ was compared to this permutation null distribution to generate an empirical *P* value for the sample *R*^2^, using Storey’s *qvalue* package. We then calculated *q*-values from these empirical *P* values, controlling for a false discovery rate of 0.05 [29]. Lastly, we constructed confidence intervals for *R*^2^ by inverting the acceptance region from the permutation test [30].

#### Validation in CBCS

We used an entirely held-out sample of 2308 women from CBCS as a validation set of Nanostring nCounter data on a codeset of 166 genes. These samples were normalized as outlined before. We used the same validation methods as in TCGA, as well using a permutation method to assess the statistical significance of predictive performance, stratified by PAM50 subtype and estrogen receptor status.

#### PAM50 subtyping

GReX in CBCS were first estimated as outlined above. We residualized the original tumor expression *E* for these imputed expression values to form a matrix of tumor expression adjusted for GReX ($\overset{\sim }{E}$). We then classified each subject into PAM50 subtypes based on both *E* and $\overset{\sim }{E}$, using the procedure summarized by Parker et al. [95, 96].

#### Survival modeling

Here, we defined a relevant event as a death due to breast cancer. We aggregated all deaths not due to breast cancer as a competing risk. Any subjects lost to follow-up were treated as right-censored observations. We estimated the association of GReX with breast cancer survival by modeling the race-stratified cause-specific hazard function of breast cancer-specific mortality, stratifying on race [97]. For a given gene *g*, the model has form

$$ {\lambda}_k(t)={\lambda}_{0k}(t){e}^{GRe{X}_g{\beta}_g+{Z}_C{\beta}_C}, $$

where *β*_*g*_ is the effect size of *GReX*_*g*_ on the hazard of breast cancer-specific mortality, *Z*_*C*_ represents the matrix of covariates (age at diagnosis, estrogen receptor status at diagnosis, tumor stage at diagnosis, and study phase), and *β*_*C*_ are the effect sizes of these covariates on survival. *λ*_*k*_(*t*) is the hazard function specific to breast cancer mortality, and *λ*_0*k*_(*t*) is the baseline hazard function. We test *H*_0_ : *β*_*g*_ = 0 for each gene *g* with Wald-type tests, as in a traditional Cox proportional hazards model. We correct for genomic inflation and bias using *bacon*, a method that constructs an empirical null distribution using a Gibbs sampling algorithm by fitting a three-component normal mixture on *Z*-statistics from TWAS tests of association [98].

Here, we consider only the 46 genes that have CV *R*^2^ > 0.01 in AA women and the 57 genes that have CV *R*^2^ > 0.01 in WW women for race-stratified survival modeling. We adjust tests for *β*_*g*_ via the Benjamini-Hochberg procedure at a false discovery rate of 0.10.

For comparison, we run a GWAS to analyze the association between germline SNPs and breast cancer-specific survival using *GWASTools* [99]*.* We use a similar cause-specific hazards model with the same covariates as in the TWAS models of association, correcting for false discovery with the Benjamini-Hochberg procedure.

#### Inspection of collider bias

To assess collider bias when conditioning for breast cancer incidence in case-only studies, such as CBCS, we test for association for the GReX of genes with breast cancer risk using iCOGs summary statistics from BCAC [37–39], using the weighted burden test identified by FUSION [14]. In summary, we compose a weighted *Z* test statistic as follows:

$$ \overset{\sim }{Z}=\frac{WZ}{{\left(W{\varSigma}_{s,s}{W}^{\prime}\right)}^{1/2}}, $$

where *Z* is the vector of *Z*-statistics from iCOGs and $W={\varSigma}_{\boldsymbol{e},s}{\varSigma}_{\boldsymbol{s},\boldsymbol{s}}^{-1}$ with **Σ**_***e,s***_ is the covariance matrix between all SNPs represented in *Z* and the gene expression of the given gene and **Σ**_*s*,*s*_ is the covariance among all SNPs.

#### Power analysis

Using *survSNP* [100], we generated the empirical power of a GWAS to detect various hazard ratios with 3828 samples with 1000 simulation replicates at a significance level of *P* = 1.70 × 10^−8^, corresponding to an FDR-adjusted *P* = 0.10. We assume an event rate of 10% and a relative allelic frequency of the risk allele of 0.1 and estimate the 90th percentile of times-to-event as a landmark time. Similarly, for genes of various cis-*h*^2^, we assessed the power of TWAS to detect various hazard ratios at *P* = 0.0096 (corresponding to FDR-adjusted *P* = 0.10) over 1000 simulation replications from the empirical distribution function of the GReX of the given gene.

## Supplementary information

Additional file 1. Supplemental Results. (DOCX 53 kb)

Additional file 2. Supplemental Figures. (DOCX 15628 kb)

Additional file 3. Supplemental Tables. (DOCX 19 kb)

Additional file 4. Review history. (DOCX 27 kb)

## Data Availability

Summary statistics eQTL results, tumor expression models, and relevant R code for training expression models in CBCS are freely available at https://github.com/bhattacharya-a-bt/CBCS_TWAS_Paper/ [101]. CBCS expression and genotype datasets analyzed in this study are not publicly available as many CBCS patients are still being followed and accordingly CBCS data is considered sensitive; the data is available from M.A.T upon reasonable request. TCGA genotype expression was accessed from the National Cancer Institute’s Genomic Data Commons Legacy Archive and TCGA expression data is available the Broad GDAC Firehose repository (https://gdac.broadinstitute.org/) with accession number phs000178.v11.p8. Functional annotation data was downloaded from ENCODE repository (DNase hypersensitive sites accession number ENSCR000EPJ and transcription factor ChIP-seq clusters with accession number wgEncodeEH001774 from: http://genome.ucsc.edu/cgi-bin/hgTrackUi?db=hg19&g=wgEncodeRegTfbsClusteredV3.)
