# Supplementary material for: A framework for transcriptome-wide association studies in breast cancer in diverse study populations
Source: Genome Biol. 2020 Feb 20;21:42. doi: 10.1186/s13059-020-1942-6 (PMC7033948; doi:10.1186/s13059-020-1942-6)
Supplement: Supplementary file 1 — Supplemental Results. (DOCX 53 kb) [file 13059_2020_1942_MOESM1_ESM.docx]

**Supplemental Results**

*Admixture and genotype principal components correlate with self-reported race*

In downstream analyses (eQTL analysis and training of predictive tumor expression), we stratify on self-reported race. We understand that self-reported race is not fully reflective of genetic ancestry. However, **Additional File 2: Figure S1** shows that self-reported race correlates strongly with population stratification in the CBCS. Furthermore, we see a linear relationship between African admixture estimates and the first genotype principal component of representative samples from the CBCS cohort that are included in the Alberta Moving Beyond Breast Cancer (AMBER) Cohort Study [1]. For all downstream eQTL analysis and tests of associations, we control for the first five principal components of the combined self-reported African American (AA) and self-reported white (WW) genotype matrix, accounting for differences in ancestry prior to stratification by self-reported race. We refer to self-reported race as race throughout this work.

*Tumor purity adjustment and overlap with healthy tissue eQTLs*

Geeleher et al. show that only a third of conventional eQTLs in bulk breast cancer tumor expression could be attributed to cancer cells in TCGA [2]. We wished to assess the extent to which this observation bore out in CBCS. For this reason, we developed an estimate of tumor purity to use as an adjustment covariate for our eQTL analysis (see **Methods**). In general, we do not see significant differences in the strength and location of significant eQTLs, as shown in comparative cis-trans plots of all eQTLs across race and adjustment for tumor purity (**Additional File 2: Figures S4 and S5**). For most genes, top eQTL regions with linkage disequilibrium (LD) support had small differences in strength of effect size (Manhattan plot for a representative gene shown in **Additional File 2: Figure S4**). Adjusting for tumor purity, at $BBFDR<0.05$ and after quality control (see **Methods**), we identified 266 cis-eQTLs and 84 trans-eQTLs in the AA sample across 36 eGenes, and 634 cis-eQTLs and 14 trans-eQTLs in the WW sample across 23 eGenes, shown in **Additional File 2: Figure S5**. All WW eGenes, adjusting for tumor purity, are in common with WW eGenes from bulk tumor expression, and 32 of 36 AA eGenes, adjusting for tumor purity, are in common with AA eGenes from bulk tumor expression. Top eQTLs for eGenes remain largely the same across adjustment for tumor purity. Summary statistics for these eQTLs are provided, as mentioned in **Availability of data and materials**. Due to limited differences when we adjust for tumor purity, all downstream analyses do not involve our computational estimate of tumor purity.

We do not observe the same difference in eQTLs across adjustment for tumor purity as in Geeleher et al [2]. The Nanostring expression data from CBCS includes only 417 genes, all of which were selected for the panel because of their involvement in breast cancer tumorigenesis, biology, or outcome disparities due to race. Furthermore, our normalization procedure involves the RUV method, which accounts for unwanted technical and biological variation, estimated from the distributions of housekeeping negative controls [3,4] with an unsupervised method. We hypothesize that the RUV method accounts for a significant percentage of the variability from cell-type heterogeneity that may confound traditional eQTL analysis in bulk tumor RNA expression. Further implementations of deconvolution algorithms specialized for expression measured for targeted panels of genes, as in Nanostring, would aid in distinguishing the source cell types or tissues for various breast tumor eQTLs. Accurate bulk expression deconvolution may also be important in future TWAS to consider sources of variation in tumor expression due to tissue heterogeneity and how deconvoluted tumor expression signals contribute to outcomes of interest.

*Local ancestry adjustments for race-specific cis-eQTLs*

For cis-eGenes identified in only one of AA or WW women, we followed up and controlled for estimated local ancestry, as inferred from source populations of Northern and Western European (CEU) and Yoruban (YRI) descent from the 1000 Genomes Project [5] (see **Methods**). Overall, we find marginal increase in the strength of association between lead SNP and cis-eGene using an estimated local ancestry-adjustment over the association measured with a genome-wide ancestry adjustment. However, we did not observe considerable harmonization of stratified cis-eQTLs across populations; in general, race-specific, local ancestry-adjusted lead cis-eQTLs in a given race-stratified sample did not show similar association in the other (**Additional File 2: Figure S6**).

It has been shown that, due to allele frequency differences between populations, the underlying genetic and eQTL architecture for complex traits may not be well-correlated across diverse populations[6,7]. Zhong et al shows that incorporating local ancestry helps to better characterize the heritability of gene expression and complex traits and accurately map genetics associations[8]. However, our local ancestry-adjusted cis-eQTLs were not well-correlated across AA and WW women. Perhaps, the persistence of this difference can be due to the simplicity of the commonly used assumption that there are two major source populations of admixture in CBCS samples (i.e. CEU and YRI). Several genetic studies into the genome-wide and local hereditary of admixed populations in the United States have shown that migratory patterns greatly inform these patterns of genetic ancestry [9,10]. Though this follow-up analysis is beyond the scope of this paper, a full cis-trans eQTL ancestry incorporating local ancestry estimates, as well as an assessment of the impact of local ancestry adjustment on the portability of our eventual predictive models of tumor expression across ancestral populations, could reveal insights into the genetic architecture of breast tumor expression heritability in admixed populations.

*Discrepancy between CBCS and TCGA platforms and source populations*

Our models showed strong cross-validation predictive performance in genes with significant cis-heritability. We also show strong predictive performance in a held-out test set from CBCS and adequate performance of our WW models in TCGA-BRCA data. We noticed a difference in EV $R^{2}$ of our predictive expression models in held-out CBCS samples and TCGA-BRCA. We believe that this difference can be attributed partly to the difference in genotyping platform between the two samples (only approximately 85% of SNPs from CBCS represented in TCGA imputed genotype data). There could also be a lack of cis-heritability of the tumor expression of a majority of genes assayed in TCGA. For example, Gusev et al. has trained models for gene expression in breast tumors in TCGA; only 8 of the 417 genes in the CBCS Nanostring panel showed significant cis-heritability in their models [11], which we downloaded from the Gusev Lab’s TWAS/FUSION repository.

We believe that predictive performance in TCGA data consistent with CBCS data is a high bar for validation due to both genotyping and RNA expression platform differences between CBCS (Oncoarray and Nanostring) and TCGA (Affymetrix 6.0 and RNAseq). Reproducible performance in both AA and WW women in our independent test set from CBCS data suggests that our models are quite robust. Follow-up studies, in which models of tumor expression are trained in TCGA RNA-seq data and validated in CBCS Nanostring data, could elucidate any discrepancies in predictive performance across platform.

*PAM50 subtype calls robust to adjustment for GReX*

Emami et al. has shown that genetically regulated tumor expression can elucidate biological mechanisms that can delineate prostate cancer subtypes [12]. Using tumor expression data in held-out CBCS, we assessed differences in PAM50 molecular subtype calls before and after adjustment by imputed GReX. We adjusted bulk TCGA tumor expression by imputed GReX and ran PAM50 subtyping on full and GReX-adjusted tumor expression [13]. Of the 2,174 samples analyzed for PAM50 subtyping, only 15 show different PAM50 subtypes after adjusting for GReX (**Additional File 2: Figure S12A**). Most of these discordant pairs (12 out of 15) are between the HER2-enriched, Luminal A, and Luminal B subtypes, all of which are relatively similar in terms of proliferation and their molecular profiles. Discordant pairs show relatively similar confidence scores (defined as $1 - P$-value of Spearman correlation to PAM50 subtype centroid), proliferation scores, and ROR-P scores [13], shown in **Additional File 2: Figure S12B-D**. The robustness of PAM50 subtype calling to GReX adjustment is consistent with our observation that only 10 of the 50 genes comprising the PAM50 gene signature were cis-heritable at $P < 0.10$ in our data set.

*Power in TWAS to detect survival associations*

Previous studies have suggested increased power in TWAS to detect smaller effect sizes in studies of disease risk [21,22]. We generated the empirical power of a GWAS to detect various hazard ratios with 3,828 samples using 1,000 simulation replicates with an event rate, risk allele frequency, landmark time and probabilities to landmark times derived from CBCS genotype data at a significance level of $P = 1.70\times{10}^{-8}$, corresponding to a FDR-adjusted $P = 0.10$[23]. Similarly, for simulated genes with various cis-$h^{2}$, we assessed the power of a hypothetical TWAS analysis to detect various gene-mediated hazard ratios at $P = 0.0096$ (corresponding to FDR-adjusted $P = 0.10$) over 1,000 simulation replications from the empirical distribution function of the GReX. It is important to note that the detectable hazard ratios at 80% for GWAS and TWAS are incomparable due to differences in units of measure. At 80% power, a GWAS with CBCS data with $N = 3,828$ is powered to detect a hazard ratio of breast cancer-specific survival of 1.88 with an addition of one alternative allele in a given SNP. At 80% power, in our study, TWAS can detect hazard ratios 1.186, 1.203, and 1.216 with the GReX of a gene with cis-$h^{2}\approx$ 0.100, 0.055, and 0.030, with respect to an increase of one standard deviation, respectively (**Additional File 2: Figure S15**).

**References**

1. Ruiz-Narváez EA, Sucheston-Campbell L, Bensen JT, Yao S, Haddad S, Haiman CA, et al. Admixture Mapping of African-American Women in the AMBER Consortium Identifies New Loci for Breast Cancer and Estrogen-Receptor Subtypes. Front Genet [Internet]. Frontiers Media SA; 2016 [cited 2019 Jun 9];7:170. Available from: http://www.ncbi.nlm.nih.gov/pubmed/27708667

2. Geeleher P, Nath A, Wang F, Zhang Z, Barbeira AN, Fessler J, et al. Cancer expression quantitative trait loci (eQTLs) can be determined from heterogeneous tumor gene expression data by modeling variation in tumor purity. Genome Biol [Internet]. BioMed Central; 2018 [cited 2019 Apr 30];19:130. Available from: https://genomebiology.biomedcentral.com/articles/10.1186/s13059-018-1507-0

3. Troester MA, Sun X, Allott EH, Geradts J, Cohen SM, Tse C-K, et al. Racial Differences in PAM50 Subtypes in the Carolina Breast Cancer Study. J Natl Cancer Inst [Internet]. Oxford University Press; 2018 [cited 2019 May 29];110:176. Available from: http://www.ncbi.nlm.nih.gov/pubmed/28859290

4. Risso D, Ngai J, Speed TP, Dudoit S. Normalization of RNA-seq data using factor analysis of control genes or samples. Nat Biotechnol [Internet]. Nature Publishing Group; 2014 [cited 2019 Mar 22];32:896–902. Available from: http://www.nature.com/articles/nbt.2931

5. Auton A, Abecasis GR, Altshuler DM, Durbin RM, Bentley DR, Chakravarti A, et al. A global reference for human genetic variation. Nature. Nature Publishing Group; 2015. p. 68–74.

6. Fang H, Hui Q, Lynch J, Honerlaw J, Assimes TL, Huang J, et al. Harmonizing Genetic Ancestry and Self-identified Race/Ethnicity in Genome-wide Association Studies. Am J Hum Genet. Elsevier BV; 2019;105:763–72.

7. Mogil LS, Andaleon A, Badalamenti A, Dickinson SP, Guo X, Rotter JI, et al. Genetic architecture of gene expression traits across diverse populations. Epstein MP, editor. PLOS Genet [Internet]. Public Library of Science; 2018 [cited 2019 Jun 13];14:e1007586. Available from: https://dx.plos.org/10.1371/journal.pgen.1007586

8. Zhong Y, Perera MA, Gamazon ER. On Using Local Ancestry to Characterize the Genetic Architecture of Human Traits: Genetic Regulation of Gene Expression in Multiethnic or Admixed Populations. Am J Hum Genet. Cell Press; 2019;104:1097–115.

9. Baharian S, Barakatt M, Gignoux CR, Shringarpure S, Errington J, Blot WJ, et al. The Great Migration and African-American Genomic Diversity. PLoS Genet. Public Library of Science; 2016;12.

10. Bryc K, Durand EY, Macpherson JM, Reich D, Mountain JL. The genetic ancestry of african americans, latinos, and european Americans across the United States. Am J Hum Genet. Cell Press; 2015;96:37–53.

11. Gusev A, Ko A, Shi H, Bhatia G, Chung W, Penninx BWJH, et al. Integrative approaches for large-scale transcriptome-wide association studies. Nat Genet [Internet]. 2016 [cited 2019 Apr 6];48:245–52. Available from: http://www.ncbi.nlm.nih.gov/pubmed/26854917

12. Emami NC, Hoffman J, Ziv E, Witte JS. Abstract 2968: Imputation of the prostate cancer transcriptome in over 230,000 men reveals novel germline-somatic interaction mechanism of cancer risk. Epidemiology [Internet]. American Association for Cancer Research; 2018 [cited 2019 Jun 10]. p. 2968–2968. Available from: http://cancerres.aacrjournals.org/lookup/doi/10.1158/1538-7445.AM2018-2968

13. Parker JS, Mullins M, Cheang MCU, Leung S, Voduc D, Vickery T, et al. Supervised risk predictor of breast cancer based on intrinsic subtypes. J Clin Oncol [Internet]. American Society of Clinical Oncology; 2009 [cited 2019 May 20];27:1160–7. Available from: http://www.ncbi.nlm.nih.gov/pubmed/19204204

14. Shimoi T, Hamada A, Yamagishi M, Hirai M, Yoshida M, Nishikawa T, et al. *PIK3CA* mutation profiling in patients with breast cancer, using a highly sensitive detection system. Cancer Sci [Internet]. Wiley-Blackwell; 2018 [cited 2019 Aug 20];109:2558–66. Available from: http://www.ncbi.nlm.nih.gov/pubmed/29906308

15. Cizkova M, Susini A, Vacher S, Cizeron-Clairac G, Andrieu C, Driouch K, et al. PIK3CA mutation impact on survival in breast cancer patients and in ERα, PR and ERBB2-based subgroups. Breast Cancer Res [Internet]. BioMed Central; 2012 [cited 2019 Aug 20];14:R28. Available from: http://www.ncbi.nlm.nih.gov/pubmed/22330809

16. Liao Y, Liao Y, Li J, Li J, Fan Y, Xu B. Polymorphisms in AURKA and AURKB are associated with the survival of triple-negative breast cancer patients treated with taxane-based adjuvant chemotherapy. Cancer Manag Res [Internet]. Dove Press; 2018 [cited 2019 Aug 20];10:3801–8. Available from: http://www.ncbi.nlm.nih.gov/pubmed/30288111

17. Vecchi M, Confalonieri S, Nuciforo P, Viganò MA, Capra M, Bianchi M, et al. Breast cancer metastases are molecularly distinct from their primary tumors. Oncogene [Internet]. 2008 [cited 2019 Jun 5];27:2148–58. Available from: http://www.ncbi.nlm.nih.gov/pubmed/17952122

18. Storr SJ, Thompson N, Pu X, Zhang Y, Martin SG. Calpain in Breast Cancer: Role in Disease Progression and Treatment Response. Pathobiology [Internet]. Karger Publishers; 2015 [cited 2019 Jun 5];82:133–41. Available from: http://www.ncbi.nlm.nih.gov/pubmed/26330354

19. Storr SJ, Zhang S, Perren T, Lansdown M, Fatayer H, Sharma N, et al. The calpain system is associated with survival of breast cancer patients with large but operable inflammatory and non-inflammatory tumours treated with neoadjuvant chemotherapy. Oncotarget [Internet]. 2016 [cited 2019 Jun 5];7:47927–37. Available from: http://www.ncbi.nlm.nih.gov/pubmed/27323818

20. Leloup L, Wells A. Calpains as potential anti-cancer targets. Expert Opin Ther Targets [Internet]. NIH Public Access; 2011 [cited 2019 Jun 5];15:309–23. Available from: http://www.ncbi.nlm.nih.gov/pubmed/21244345

21. Wu L, Shi W, Long J, Guo X, Michailidou K, Beesley J, et al. A transcriptome-wide association study of 229,000 women identifies new candidate susceptibility genes for breast cancer. Nat Genet [Internet]. NIH Public Access; 2018 [cited 2019 Apr 25];50:968–78. Available from: http://www.ncbi.nlm.nih.gov/pubmed/29915430

22. Gusev A, Mancuso N, Won H, Kousi M, Finucane HK, Reshef Y, et al. Transcriptome-wide association study of schizophrenia and chromatin activity yields mechanistic disease insights. Nat Genet [Internet]. Nature Publishing Group; 2018 [cited 2019 Apr 6];50:538–48. Available from: http://www.nature.com/articles/s41588-018-0092-1

23. Owzar K, Li Z, Cox N, Jung S-H. Power and Sample Size Calculations for SNP Association Studies With Censored Time-to-Event Outcomes. Genet Epidemiol [Internet]. John Wiley & Sons, Ltd; 2012 [cited 2019 Jun 4];36:538–48. Available from: http://doi.wiley.com/10.1002/gepi.21645
