# Supplementary material for: A framework for transcriptome-wide association studies in breast cancer in diverse study populations
Source: Genome Biol. 2020 Feb 20;21:42. doi: 10.1186/s13059-020-1942-6 (PMC7033948; doi:10.1186/s13059-020-1942-6)
Supplement: Supplementary file 2 — Supplemental Figures. (DOCX 15628 kb) [file 13059_2020_1942_MOESM2_ESM.docx]

Supplemental Figures


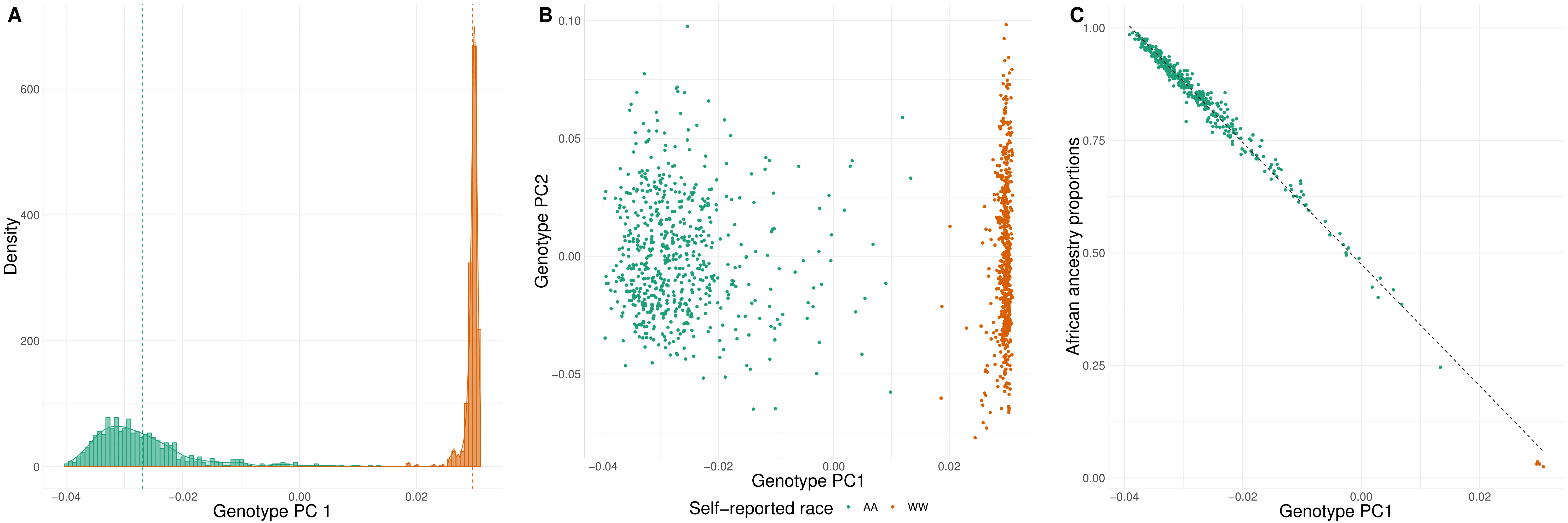


**Figure S1**: (A) Density plot of first principal component of genotype matrix, colored by self-reported race. (B) PCA plot of first principal component of genotype matrix (X-axis) and second principal component of genotype matrix (Y-axis), colored by self-reported race. (C) Plot of genotype PC1 (X-axis) against African admixture ancestry estimates from the Alberta Moving Beyond Breast Cancer (AMBER) Study Cohort (Y-axis). The sample plotted here is an intersection of patients from CBCS and the AMBER cohort.


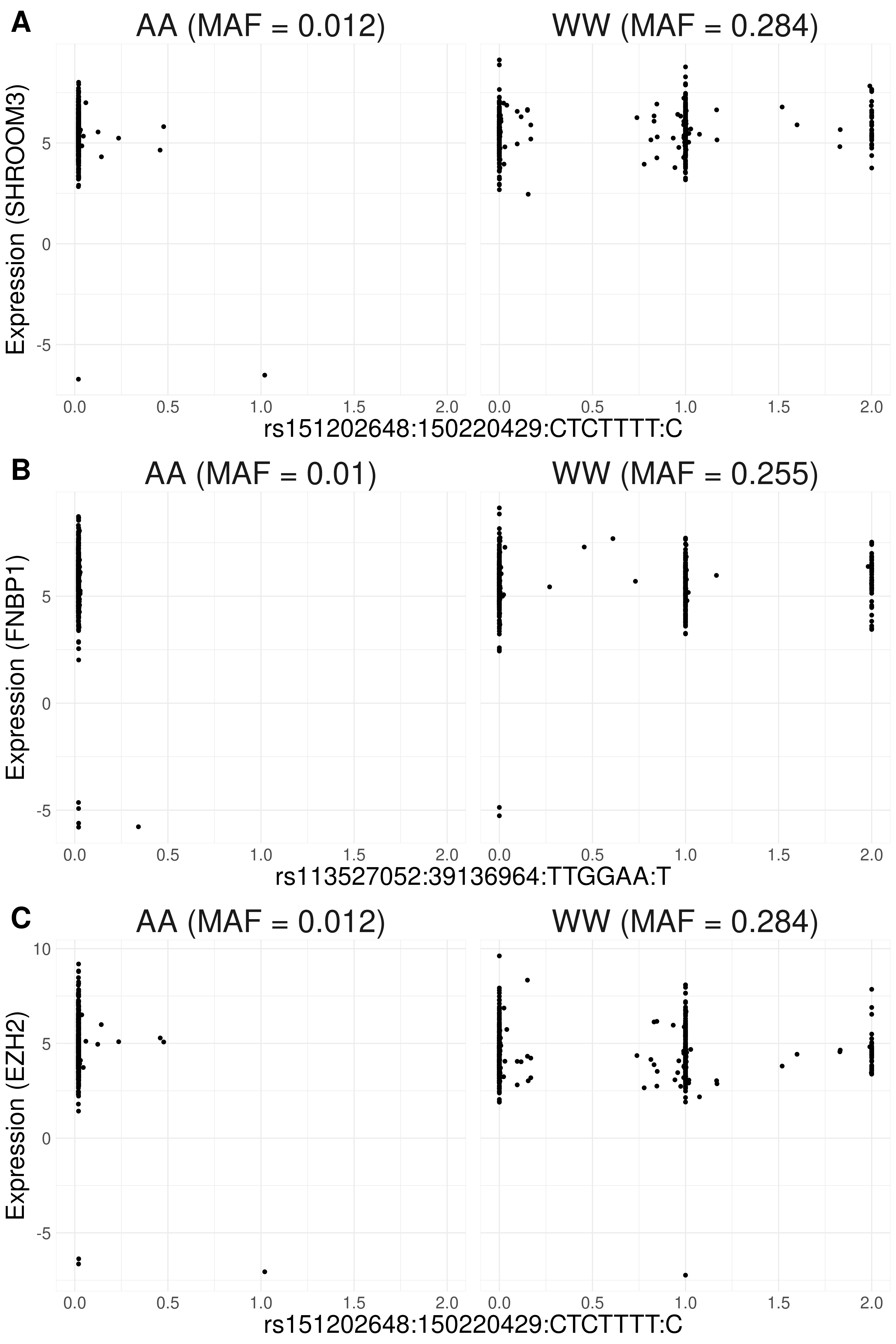


**Figure S2**: Plots of eGene expression (Y-axis) over dosage (X-axis) of associated eSNPs for three sample eQTLs that failed quality control.


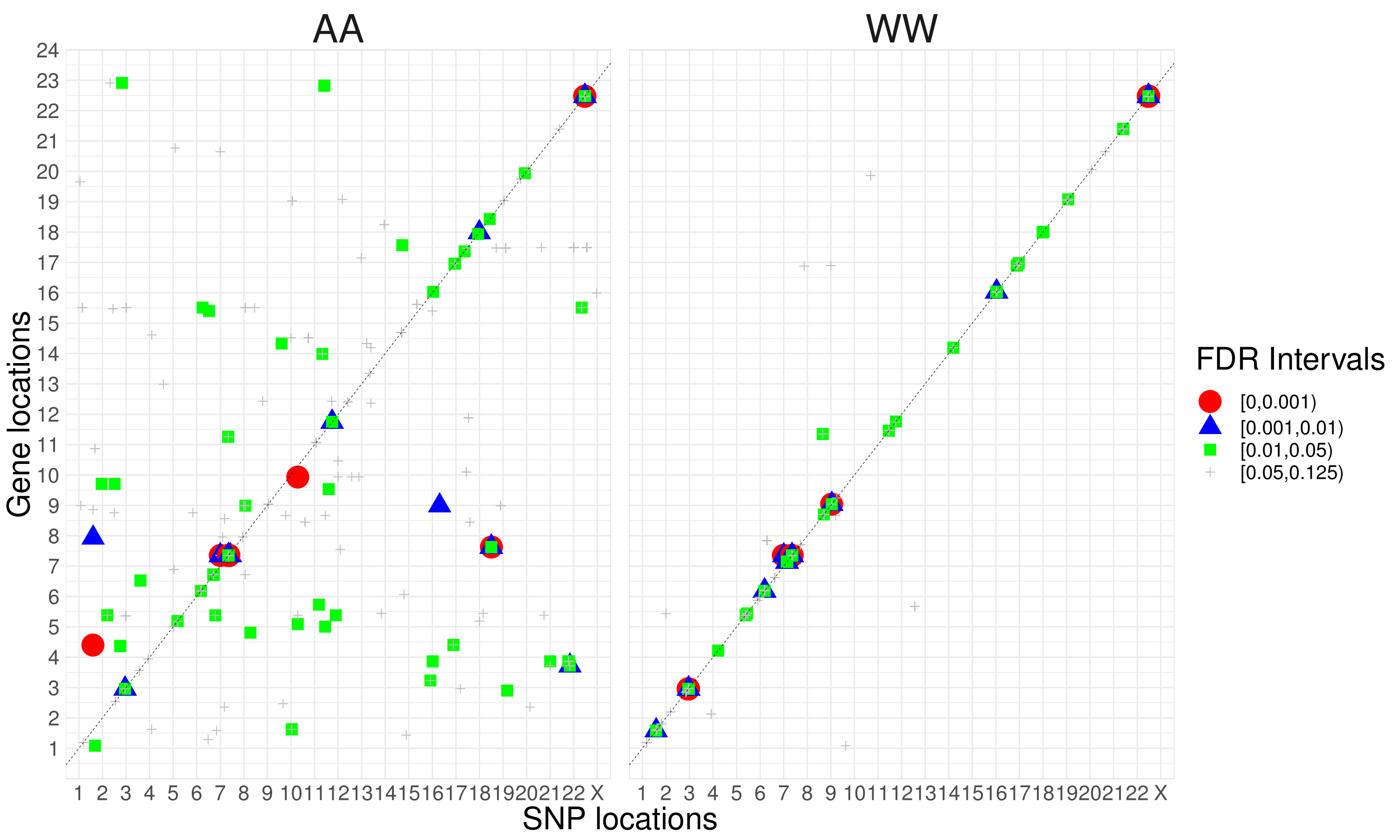


**Figure S3**: Cis-trans plot of race-stratified eQTL analyses, AA on the left and WW on the right. Each point represents an eQTL with $BBFDR<0.125$ with the location of the 5’ end of the corresponding eGenes on the $Y$-axis and the genomic location of the corresponding eSNP on the $X$-axis. A 45-degree line is provided as a reference for cis-eQTLs.


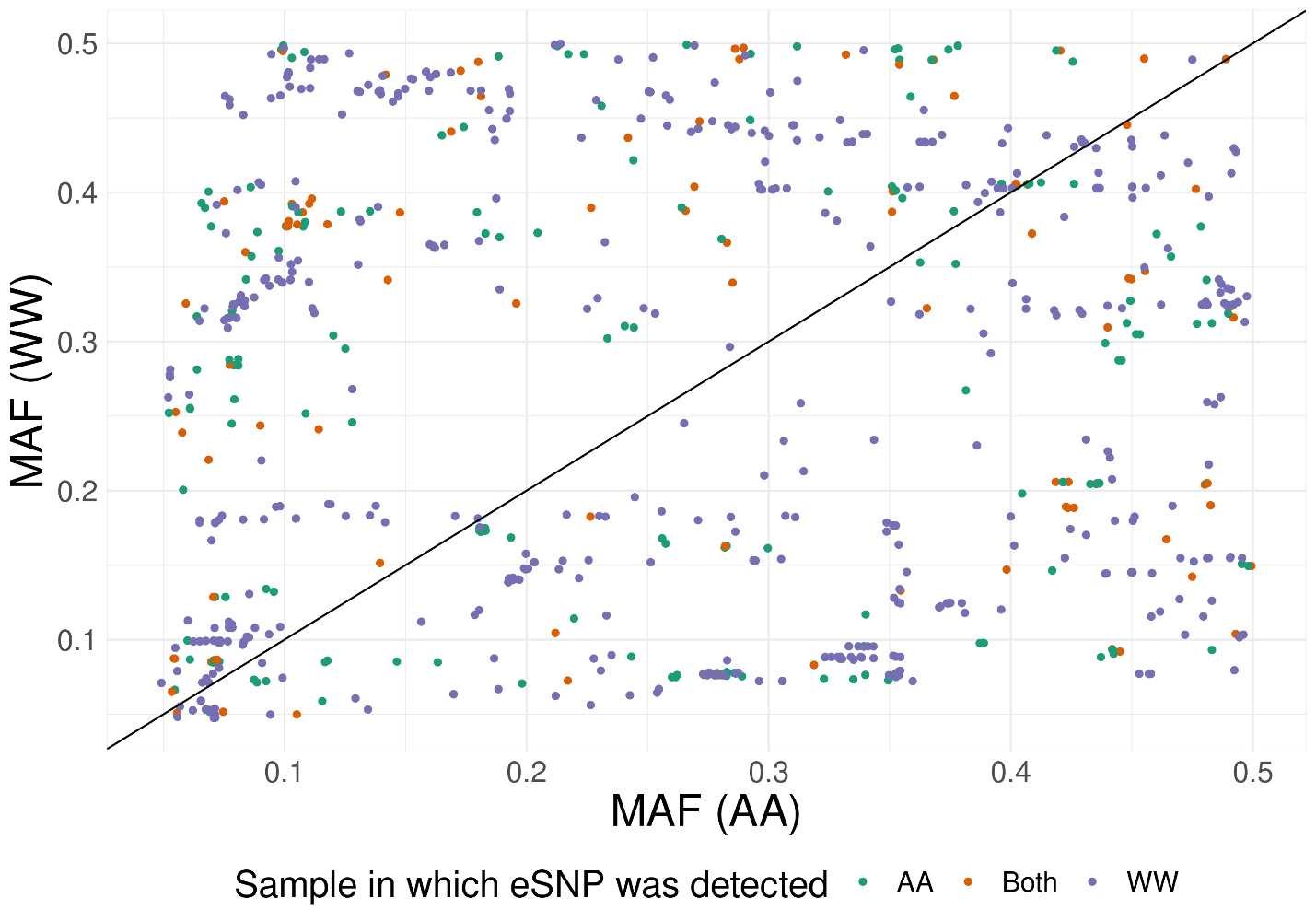


**Figure S4**: Scatter plot of minor allele frequencies (MAF) of all significant eSNPs ($BBFDR<0.05$) in either the AA or WW sample, with the MAF in the AA sample on the X-axis and in the WW sample on the Y-axis. Points are colored by the sample in which the eSNP was detected. The 45-degree line is provided for reference.


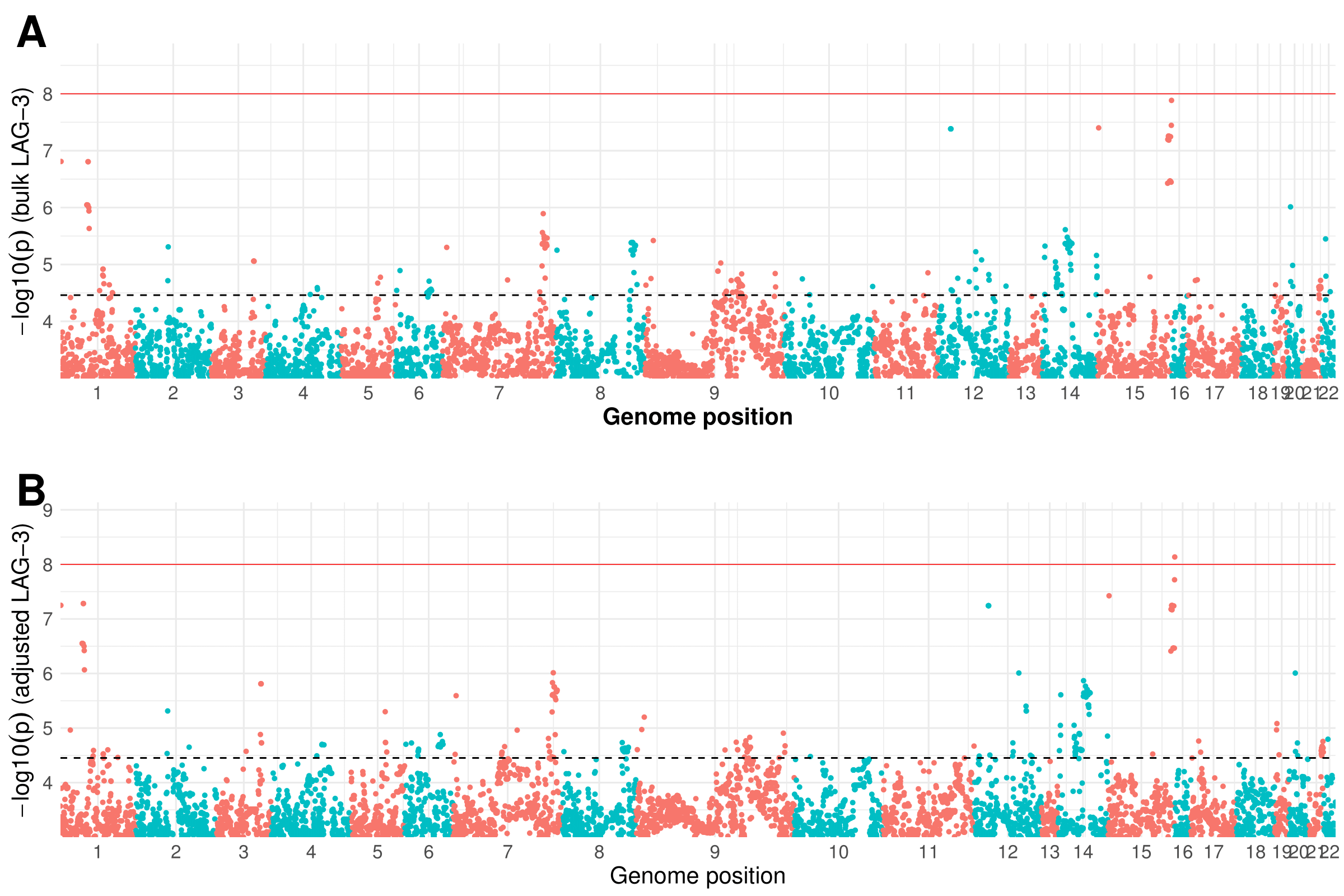


**Figure S5**: Example Manhattan plots for eQTL analysis in bulk tumor LAG-3 expression (A) and tumor purity-adjusted LAG-3 expression (B) in WW women. Red line represents a genome-wide significance threshold of $P = 1\times{10}^{-8}$ and the dotted black line corresponds to $BBFDR < 0.05$.


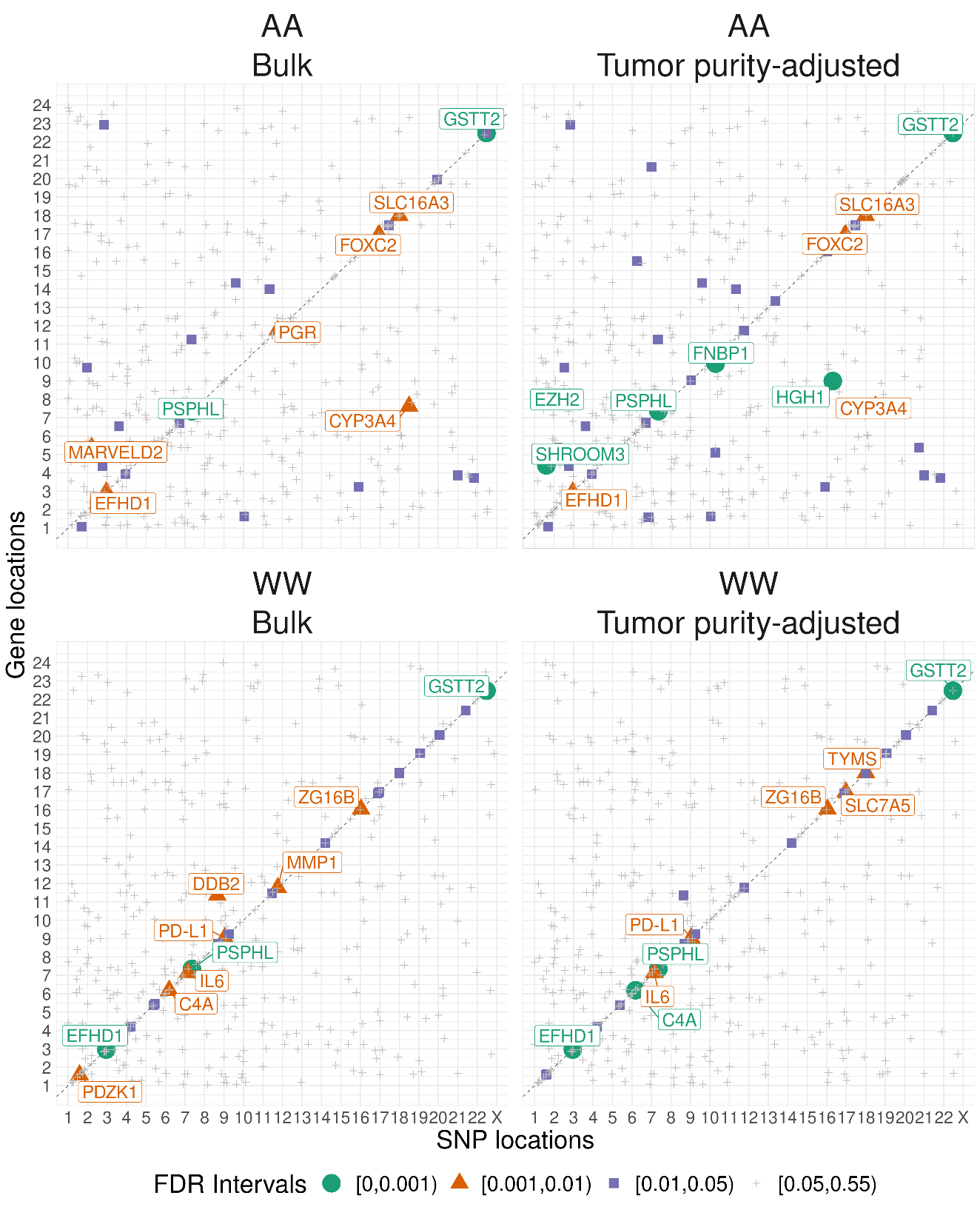


**Figure S6**: Cis-trans plots, as in **Supplementary Figure 3**, across self-identified race (top to bottom) and across adjustment for tumor purity (eQTLs in bulk tumor expression on left and eQTLs in tumor purity-adjusted expression on left)


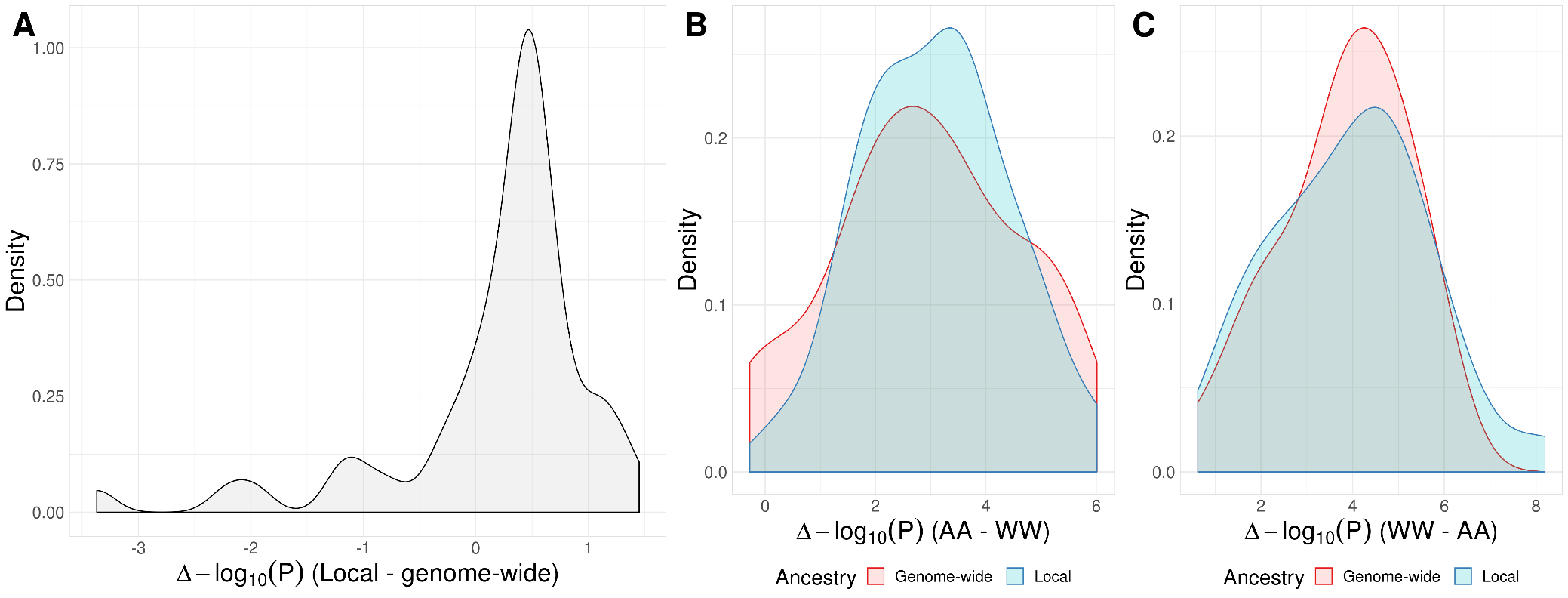


**Figure S7**: Impact of local ancestry adjustment on cis-eQTLs. (A) Kernel density plot of difference in $-{log}_{10} P$-values for lead cis-eQTLs identified with local ancestry adjustments and genome-wide ancestry adjustments. (B) Kernel density plot of difference in $-{log}_{10} P$-values of association of eQTLs between AA and WW women with genome-wide ancestry adjustment (red) and local ancestry adjusted (blue) for lead eQTLs identified for AA-specific cis-eGenes. (C) Kernel density plot of difference in $-{log}_{10} P$-values of association of eQTLs between WW and AA women with genome-wide ancestry adjustment (red) and local ancestry adjusted (blue) for lead eQTLs identified for WW-specific cis-eGenes.


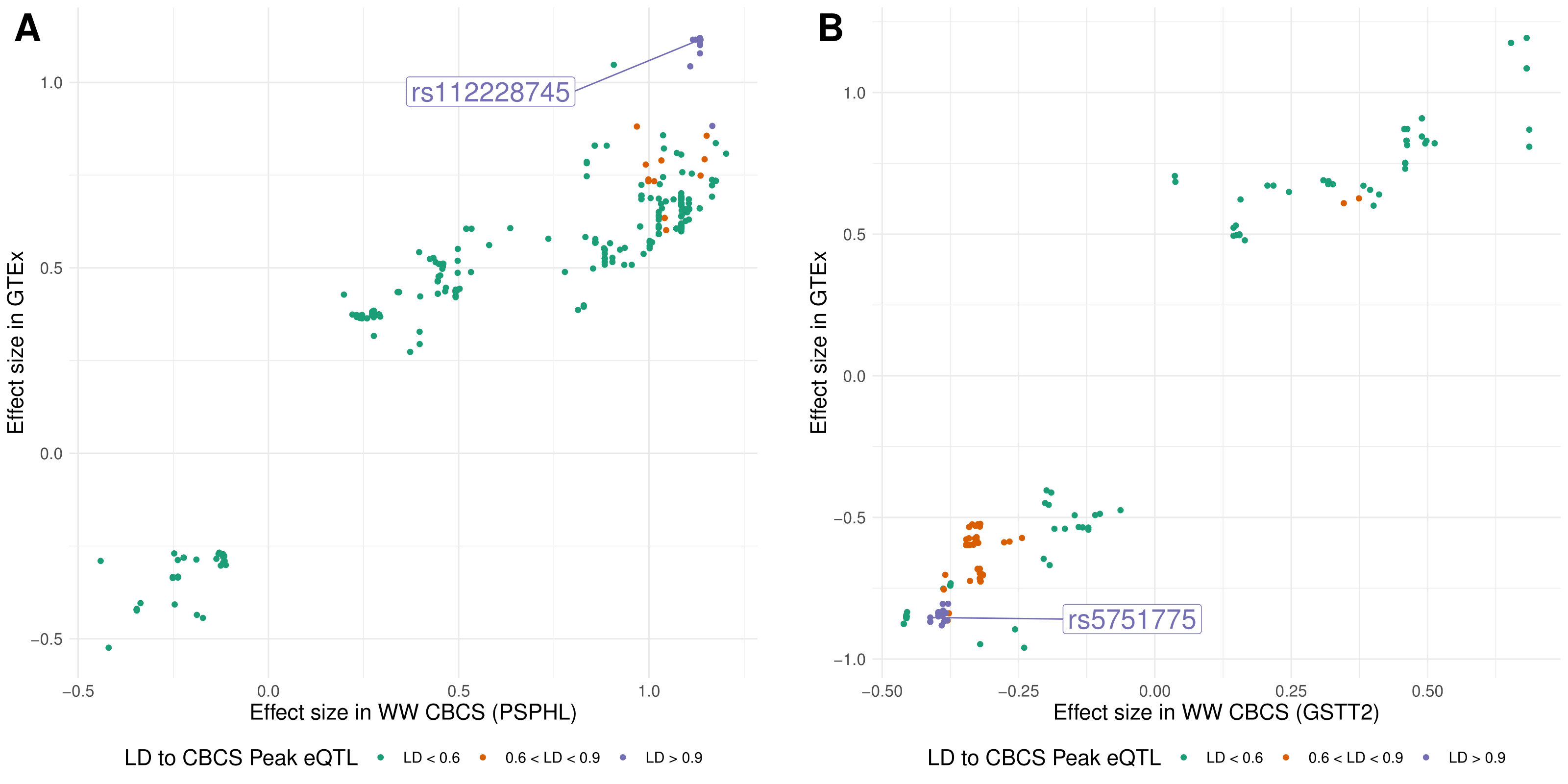


**Figure S8**: Each point represents a significant eQTL for PSPHL (A) and GSTT2 (B) found in both GTEx and the CBCS WW sample, colored by the strength of linkage disequilibrium to the top eSNP in CBCS. Absolute effect size of significant eQTLs in WW CBCS is plotted on the X-axis and absolute effect size of significant eQTLs in GTEx multiplied by the sign of the effect size in CBCS is plotted on the Y-axis.


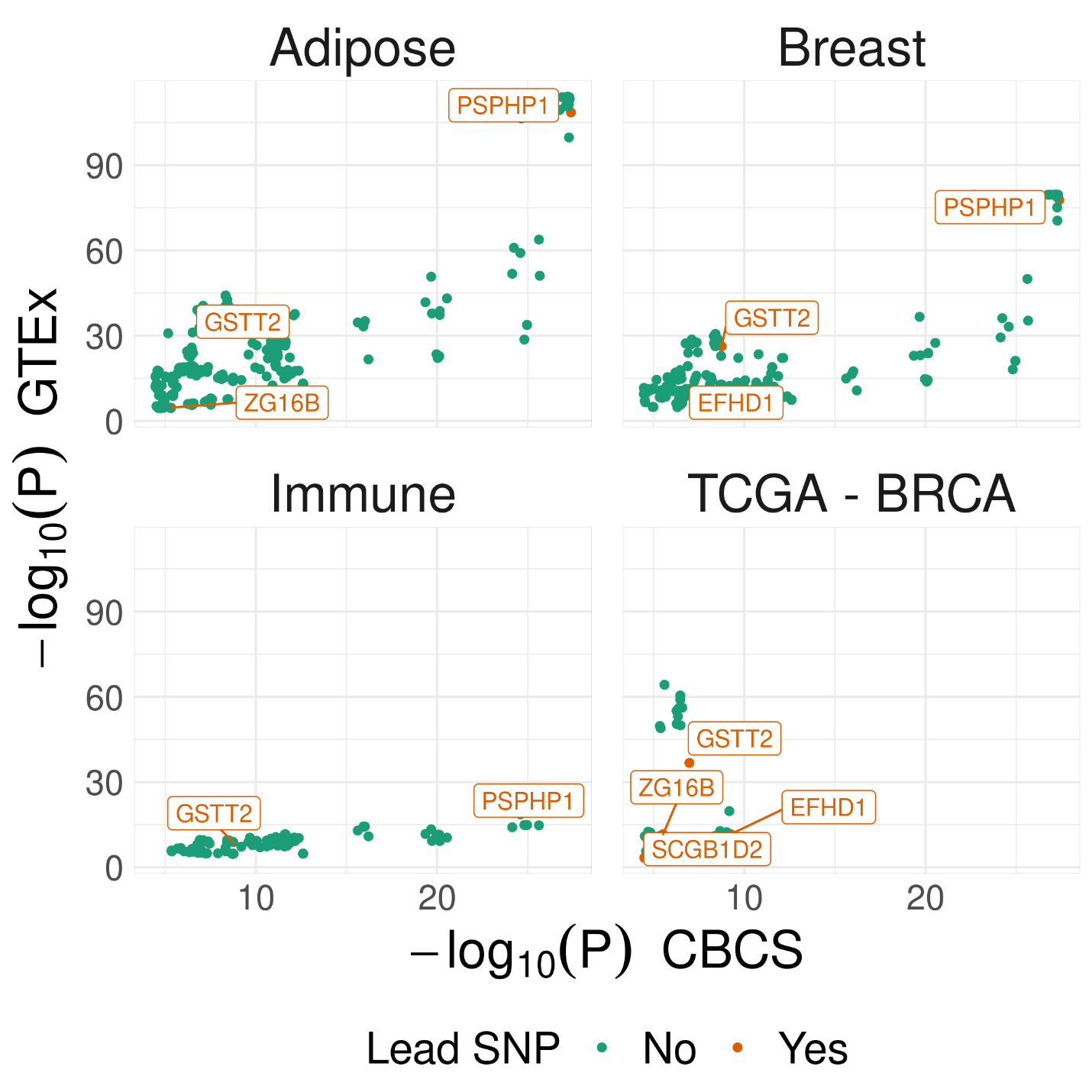


**Figure S9**: Overlap of WW CBCS cis-eQTLs in GTEx and TCGA-BRCA. Each point represents a given cis-eSNP-eGene pair (cis-eQTL), with the ${log}_{10} P$-value of the association in CBCS on the X-axis and the ${-log}_{10} P$-value of the association in the external dataset on the Y-axis. Each cis-eQTL that is colored orange and labelled is the lead cis-eSNP in CBCS (i.e. the lowest $P$-value for that eGene in CBCS).


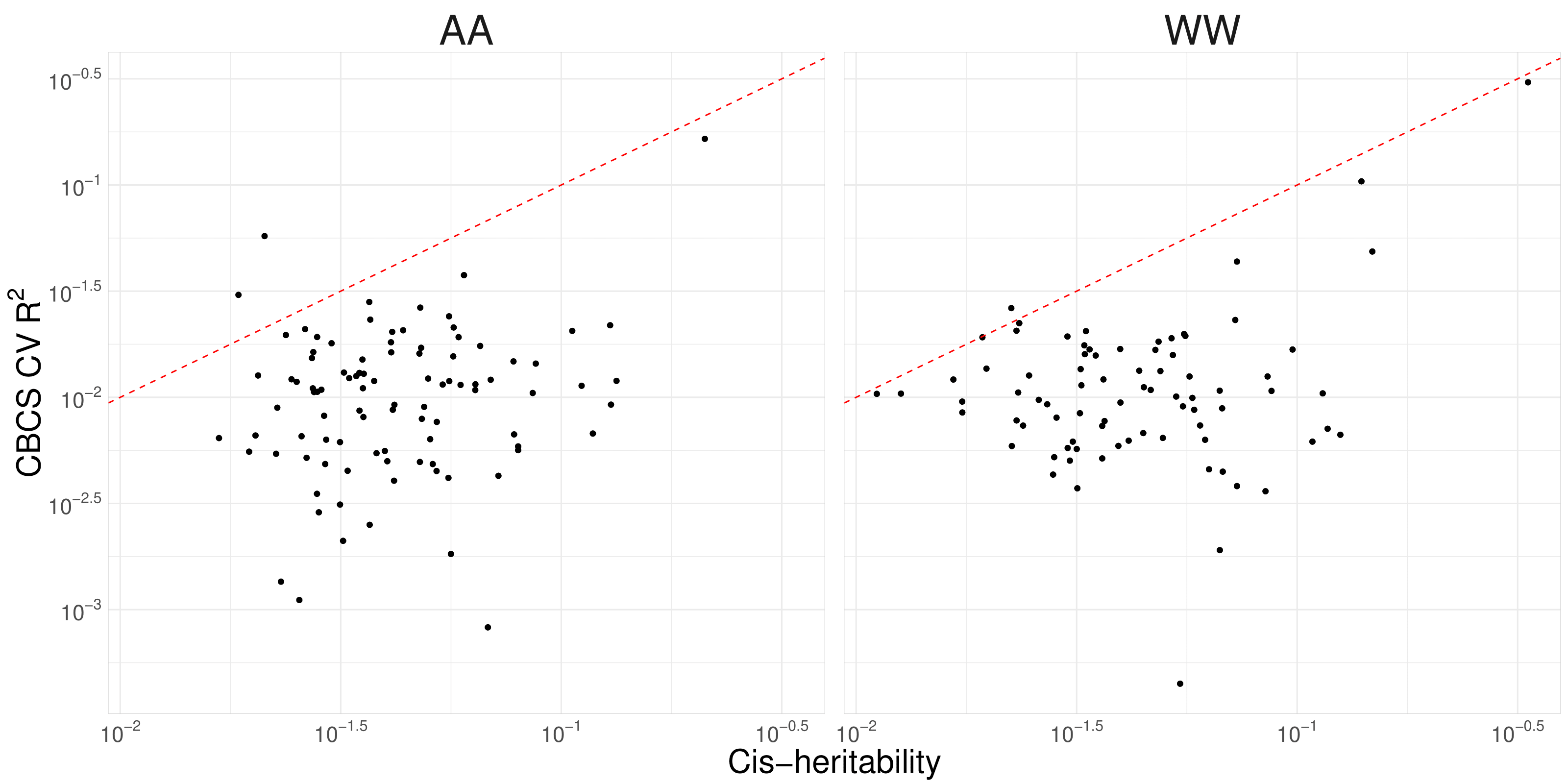


**Figure S10**: Comparison of cis-$h^{2}$ estimates (X-axis) and cross-validation $R^{2}$ (Y-axis) for each gene with likelihood ratio test $P < 0.10$ for cis-$h^{2} = 0$ across AA and WW women in CBCS training set. The 45-degree line (i.e. $Y=X$) is provided for reference in red.


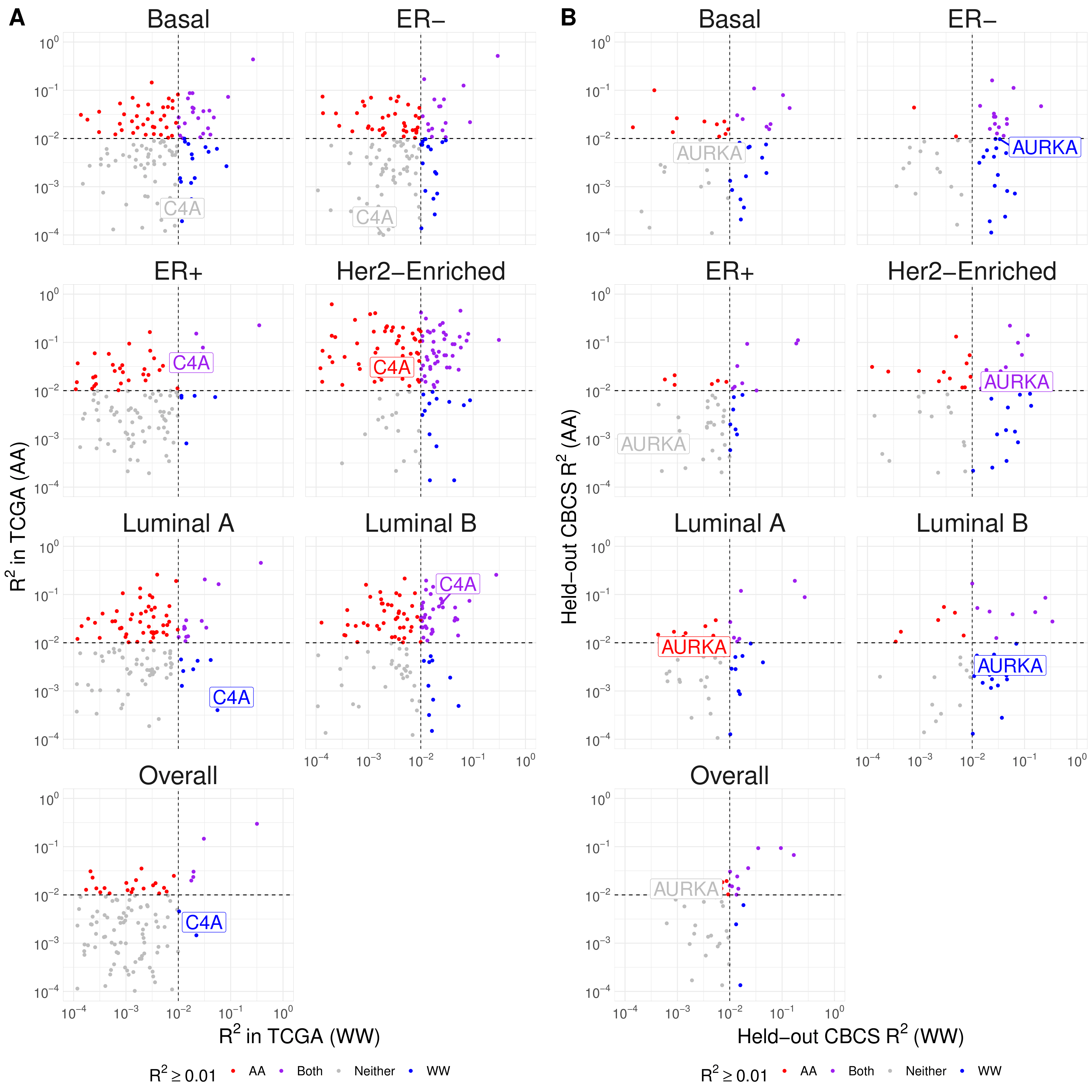


**Figure S11**: Comparison of prediction $R^{2}$ across race, stratified by PAM50 molecular subtype and estrogen receptor status in TCGA (A) and CBCS (B). Squared Spearman correlation in WW (X-axis) and AA (Y-axis) for each of the available genes are plotted. Note that both scales are logarithmic. Dotted lines represent $R^{2} = 0.01$. Colors represent the model with which a given gene can be predicted at cross-validation $R^{2} > 0.01$. A representative gene with variable $R^{2}$ across subtypes is labelled.


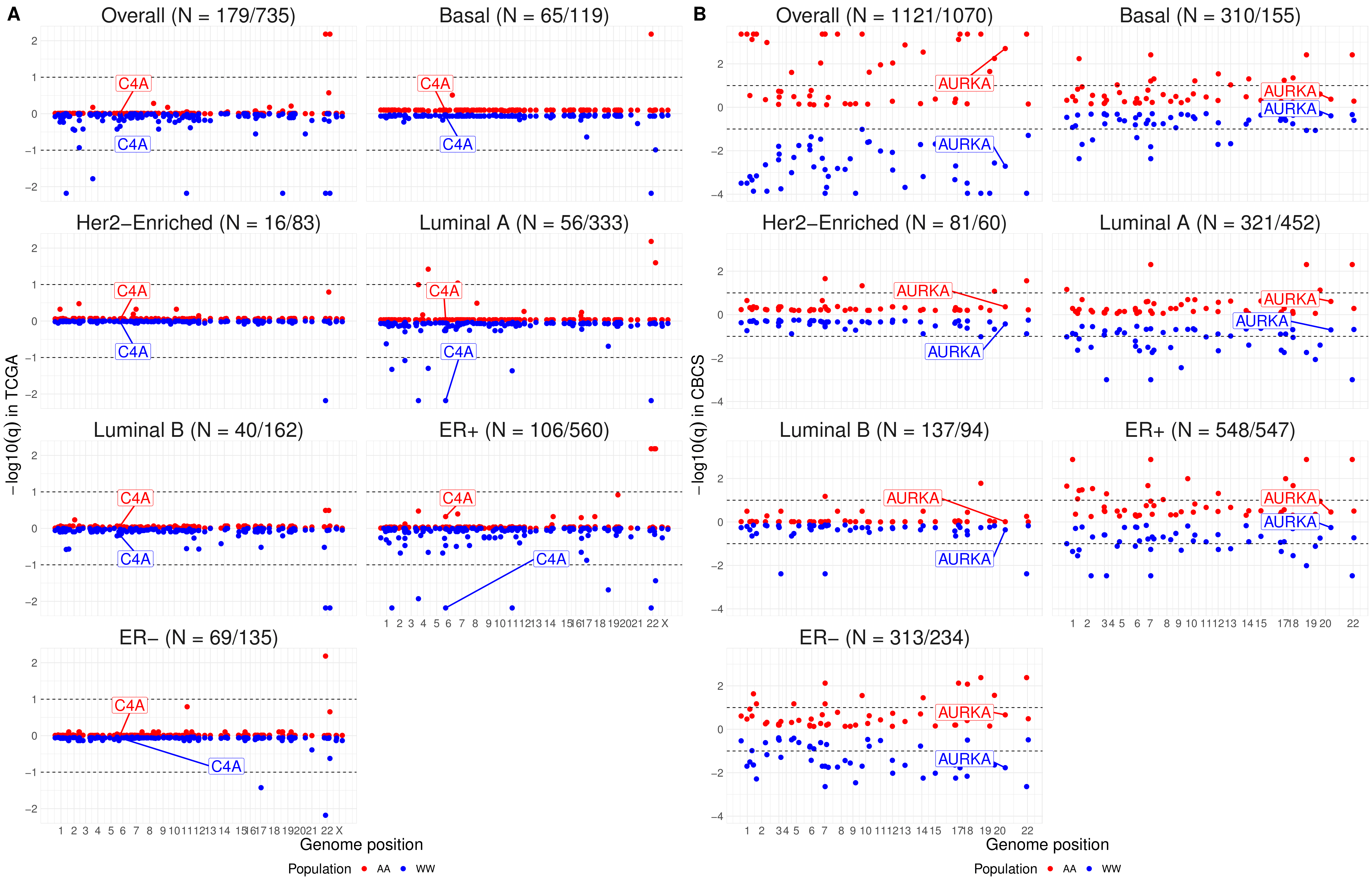


**Figure S12**: Storey’s − log10 q-values from P-values of permutation tests over 10,000 permutations to assess significance of external validation $R^{2}$ in TCGA (A) and held-out CBCS (B). Dotted lines represent q = 0.10. Sample sizes are provided in the form (AA/WW). A representative gene with variable permutation q-value across subtype is labelled.


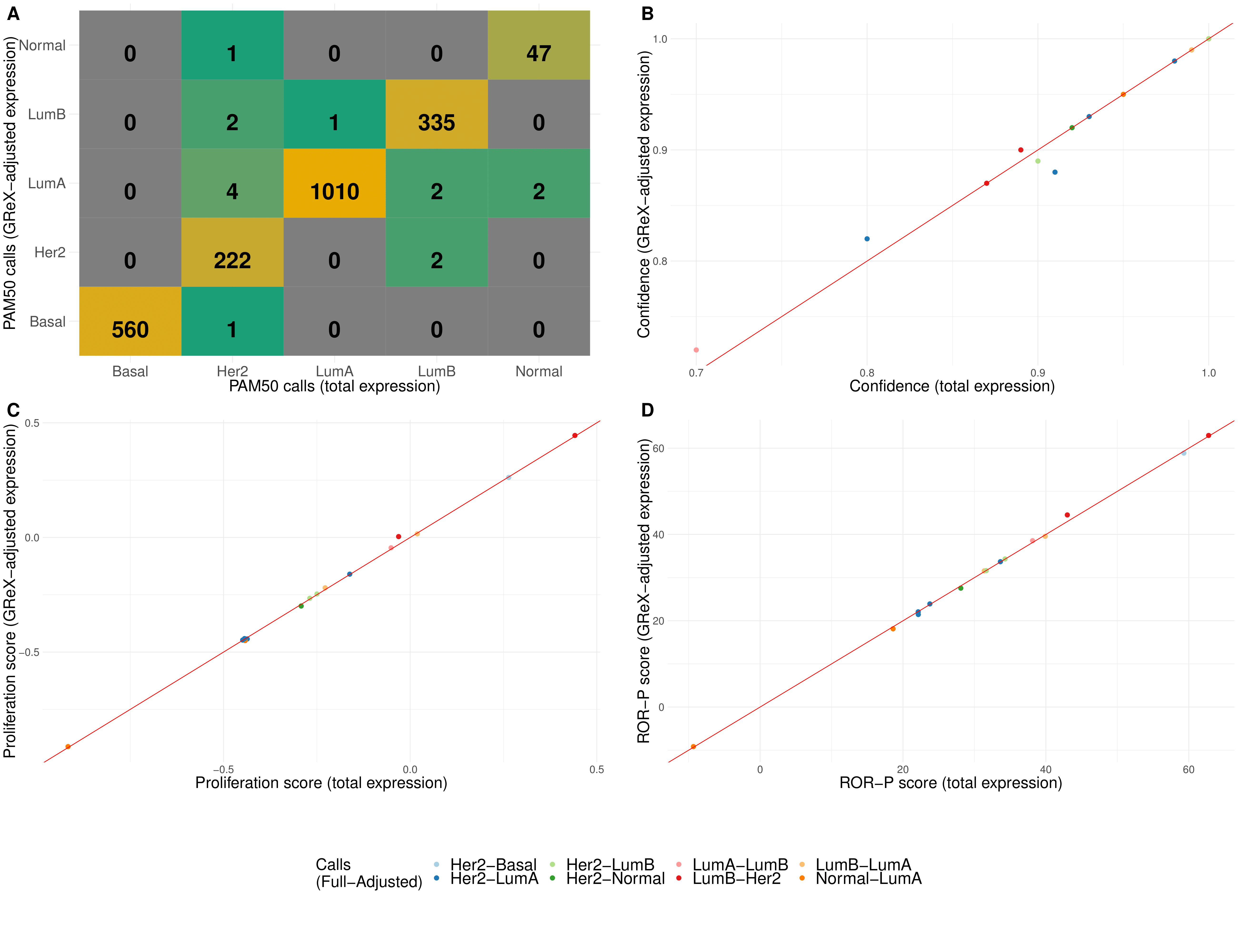


**Figure S13**: (A) Confusion matrix of PAM50 molecular subtype calls in held-out CBCS derived from full, unadjusted tumor expression(X-axis) and tumor expression adjusted for GReX (Y-axis) (B,C,D) PAM50 confidence (B), proliferation (C), and ROR-P (D) scores across adjustment for GReX. Points represent discordant calls across adjustment, colored by calls before and after adjustment. Red line provides the 45-degree regression line for reference. PAM50 confidence is defined as $1-P$-value of a sample's Spearman correlation test to the PAM50 centroid. The proliferation and ROR-P scores are linear combinations of a sample's distances to all five PAM50 centroids and are measures of a tumor's proliferation and risk of relapse.


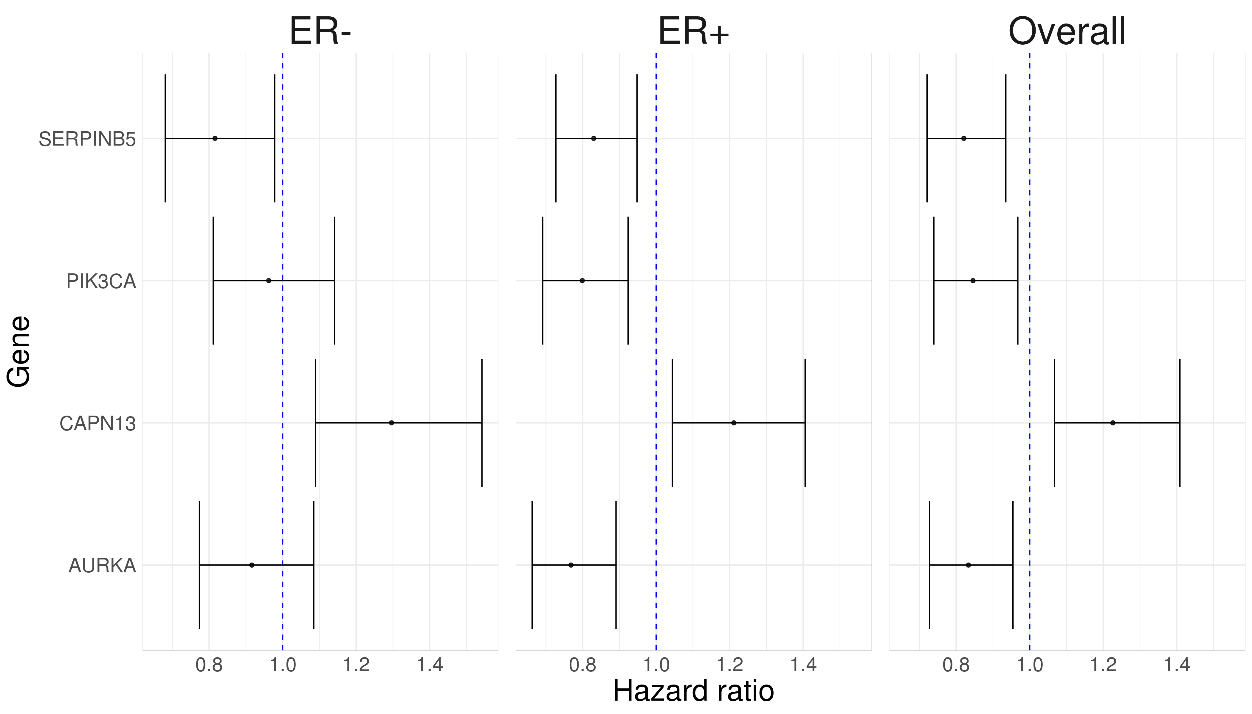


**Figure S14**: Caterpillar plots for hazard ratio of breast cancer-specific survival in AA women for an increase of one standard deviation of GReX across models unadjusted for estrogen receptor subtype and stratifying for estrogen receptor subtype.


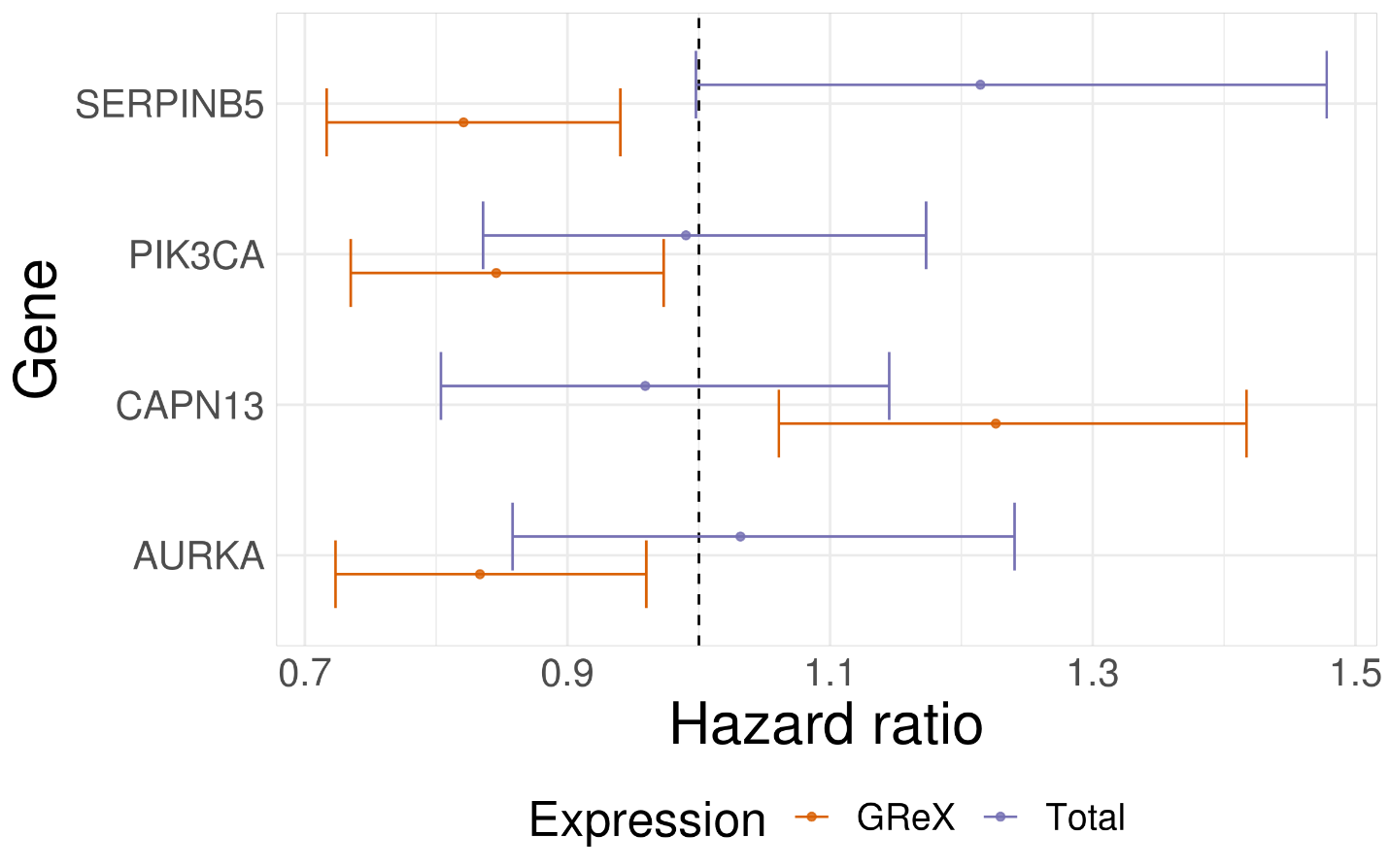


**Figure S15**: Hazard ratios and 95% confidence intervals, adjusted for false discovery via Benjamini-Hochberg, as estimated from breast cancer-specific Cox models in AA women. Association with total expression (purple) and GReX (orangle) of 4 TWAS-detected genes are compared.


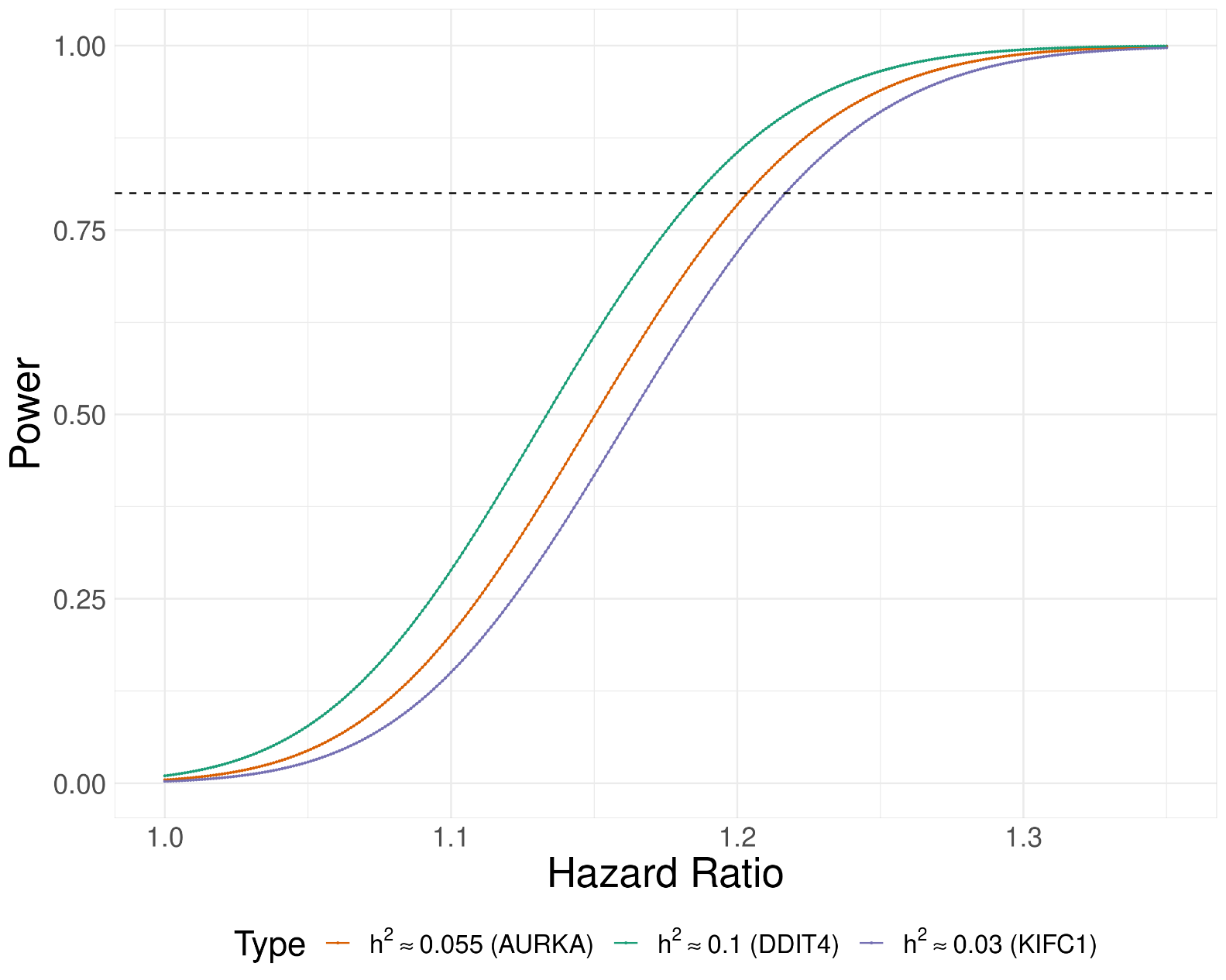


**Figure S16**: Comparison of power of TWAS in CBCS sample of $N = 3,828$ and 348 breast cancer-

specific deaths. Power (Y-axis) to detect a given hazard ratio (X-axis) is plotted. Curves correspond to genes of varying cis-$h^{2}$: *DDIT4* (green) has high $h^{2}$ across AA and WW, *AURKA* (orange) has average $h^{2}$ across AA and WW, and *KIFC1* (purple) has the lowest $h^{2}$ across AA and WW. Power calculations are derived from 1,000 re-samplings of the empirical distribution function of the GReX of a given gene. Dotted line represents 80% power.

**Figure S17**: Modified from Paternoster et al. Directed acyclic graph that shows how collider bias is introduced (grey path) in case-only studies. Here, in this case-only study, we condition on breast cancer incidence, which may open up a potential collider bias with unmeasured confounders in the measure of association between the GReX of a gene and breast cancer survival.

Genetic variant

GReX of gene

Breast cancer survival

Unmeasured confounders

Breast cancer incidence

+

-

+


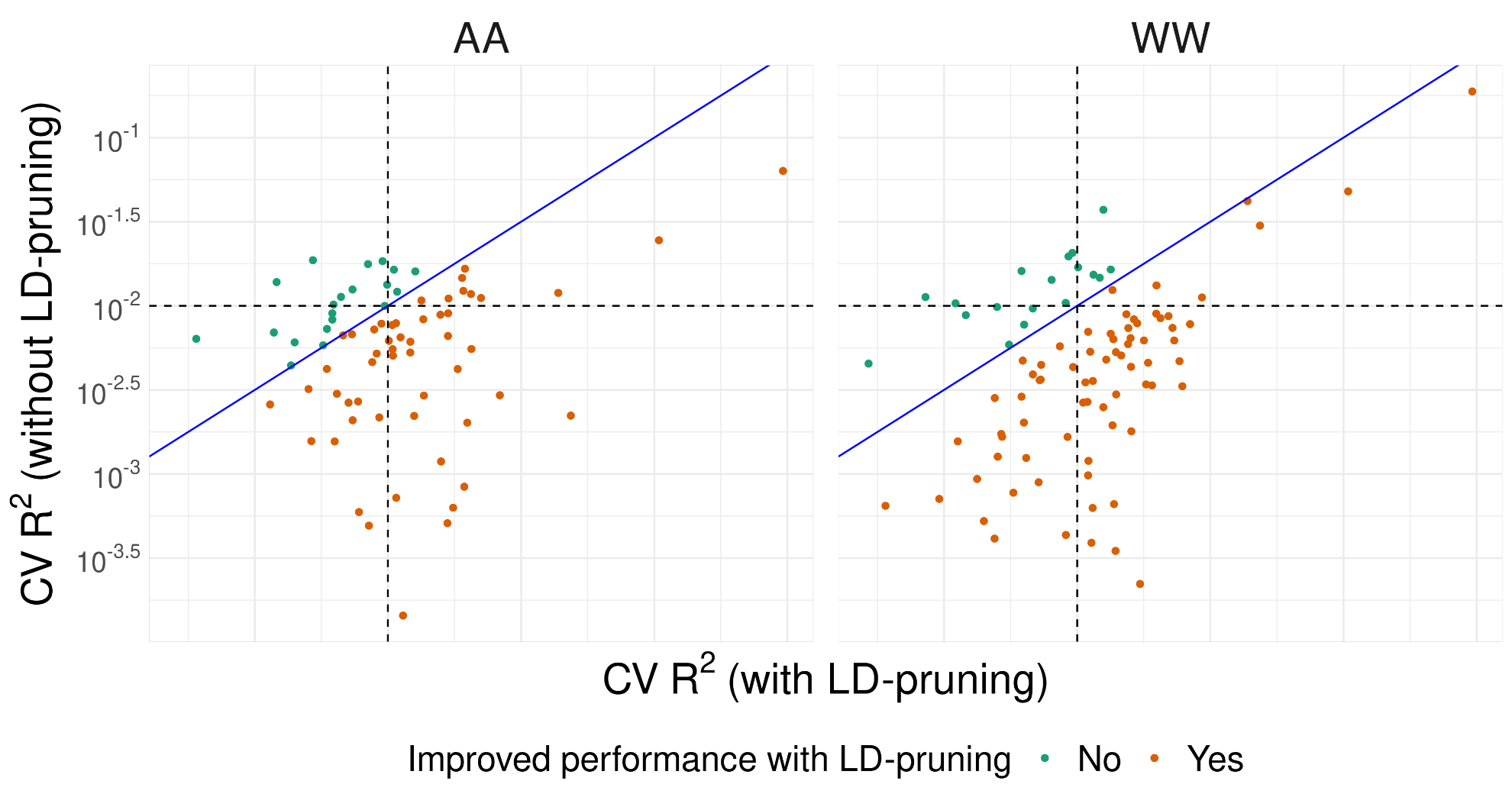


**Figure S18**: For genes with cis-$h^{2}$with $P<0.10,$ cross-validation $R^{2}$ with (X-axis) and without (Y-axis) LD-pruning of genotype design matrix. Points are colored orange if there is increased CV $R^{2}$ with LD-pruning. The blue line gives the 45-degree line and the dotted black lines show thresholds for $R^{2}=0.01$, for reference.
