# Supplementary material for: A framework for transcriptome-wide association studies in breast cancer in diverse study populations
Source: Genome Biol. 2020 Feb 20;21:42. doi: 10.1186/s13059-020-1942-6 (PMC7033948; doi:10.1186/s13059-020-1942-6)
Supplement: Supplementary file 3 — Supplemental Tables. (DOCX 19 kb) [file 13059_2020_1942_MOESM3_ESM.docx]

| **Functional enrichment**^a^ | **eQTLs in AA (343 total eQTLs)**^b^ | **eQTLs in WW (706 total eQTLs)**^b^ |
| --- | --- | --- |
| **DNAseI cleavage hotspots**  (MCF-7 cells)  Number of sites: 291,824 | *Observed number of eQTLs*: 6  *Mean expected number of eQTLs*: 0.54  *Fisher’s test*: 5.98, (0.721, 276)  $P= 0.123$ | *Observed number of eQTLs*: 20  *Mean expected number of eQTLs*: 0.71  *Fisher’s test*: 19.9, (3.18, 827)  $P = 1.9\times{10}^{-5}$* |
| **ESR1 binding sites**  (T-47D cells)  Number of sites: 10,634 | *Observed number of eQTLs*: 1  Mean expected number of eQTLs: 0.31  *Fisher’s test*: Inf (0.025, Inf)  $P= 1$ | *Observed number of eQTLs*: 1  *Mean expected number of eQTLs*: 0.42  *Fisher’s test*: Inf, (0.026, Inf)  $P=1$ |
| **Any transcription binding site**  (T-47D cells)  Number of sites: 132,022 | *Observed number of eQTLs*: 3  *Mean expected number of eQTLs*: 0.83  *Fisher’s test*: 2.99, (0.239, 158)  $P = 0.624$ | *Observed number of eQTLs*: 15  Mean expected number of eQTLs: 2.11  *Fisher’s test*: 7.49, (1.73, 67.9)  $P= 0.002$* |

**Table S1:** Functional enrichment analysis of eQTLs across (a) functional annotations for DNAaseI cleavage hotspots in MCF-7 breast cancer cells, ESR1 binding site in T-47D breast cancer cells, and any transcription binding site in T-47D cancer cells. The total number of functionally enriched sites for each analysis is provided. (b) The observed number of eQTLs within a 1kb window of a functional annotation is given, with enrichment statistics, mean expected number of eQTLs within a 1kb window of a functional annotation as estimated over 10,000 permutations, odds ratio (OR) between observed and mean expected number of eQTLs (over 10,000 permutations) overlapped in functional annotations, an associated 95% confidence interval, and a two-sided $P$-value for enrichment or depletion. Associations with (*) are those that are significant at a Bonferroni-corrected significance level of 0.05 across these 6 tests.

|  | | **AA** | | **WW** | | |  |
| --- | --- | --- | --- | --- | --- | --- | --- |
| Training/test set | **All genes**  **Mean** $\boldsymbol{R}^{\boldsymbol{2}}$ $\boldsymbol{(25\%, 75\%)}$ | | **Prioritized genes**  **Mean** $\boldsymbol{R}^{\boldsymbol{2}}$ $\boldsymbol{(25\%, 75\%)}$ | | **All genes**  **Mean** $\boldsymbol{R}^{\boldsymbol{2}}$ $\boldsymbol{(25\%, 75\%)}$ | **Prioritized genes**  **Mean** $\boldsymbol{R}^{\boldsymbol{2}}$ $\boldsymbol{(25\%, 75\%)}$ | |
| CBCS training set  Sample size: 628 AA, 571 WW | 0.012 (0.007,0.014)  417 genes | | 0.016 (0.006,0.016)  81 genes | | 0.012 (0.007,0.014)  417 genes | 0.016 (0.006,0.016)  100 genes | |
| Held-out CBCS test set  Sample size: 1121 AA, 1070 WW | 0.007 (0.002,0.008)  166 genes | | 0.013 ($8.1\times{10}^{-4}$,0.013)  50 genes | | 0.008 (0.002,0.010)  166 genes | 0.014 (0.002,0.010)  50 genes | |
| TCGA-BRCA test set  Sample size: 179 AA, 735 WW | 0.006 ($5.1\times{10}^{-4}$,0.006)  412 genes | | 0.009 ($6.0\times{10}^{-4},0.007)$  149 genes | | 0.002 ($1.0\times{10}^{-4},0.002)$  412 genes | 0.005 ($1.5\times{10}^{-4},0.002)$  149 genes | |

**Table S2**: Mean cross-validation or external validation $R^{2}$ across CBCS training set, held-out CBCS test set, and TCGA-BRCA test set. The 25% and 75% quantiles are provided in parentheses with the number of genes with the number of genes considered for these sample statistics. Note that here we define a prioritized gene as one with cis-$h^{2}\geq0$ with $P<0.1$ in the training set.
