# Supplementary material for: A framework for transcriptome-wide association studies in breast cancer in diverse study populations
Source: Genome Biol. 2020 Feb 20;21:42. doi: 10.1186/s13059-020-1942-6 (PMC7033948; doi:10.1186/s13059-020-1942-6)
Supplement: Supplementary file 4 — Review history. (DOCX 27 kb) [file 13059_2020_1942_MOESM4_ESM.docx]

Review History

**First round of review**

**Reviewer 1**

**Are you able to assess all statistics in the manuscript, including the appropriateness of statistical tests used?**

No, I do not feel adequately qualified to assess the statistics.

**Comments to author:**

Bhattacharya et al performed an integrative analysis of the eQTLs, gene expression predication and association with patient survival based on a large breast cancer cohorts CBCS with nearly equal representation of African American and White patients, and compared the results to TCGA. This integrative transcriptome-wide analysis (TWAS) allows the authors to uncover the dramatic difference between the two races in, e.g. the amount of trans versus cis-eQTLs, performance of gene expression prediction by germline variations, genes associated with patient survival, patterns in breast cancer subtypes, etc. This study underscores the importance of incorporating race or ancestry into TWAS analysis. Overall, this is an interesting and well-written manuscript. The authors should address the following comments in order to improve the quality of manuscript.

1.     The authors should describe the sample size (AA and WW) in Results - Race specific germline eQTL analysis. According to Methods, the authors analyzed 5,989,134 germline SNPs from 3,828 samples (1,865 AA and 1,963 WW). However, the gene expression data (in 406 genes) are available only in 1,388 samples. It's not clear how the authors performed eQTL analysis with many of genotype-expression unpaired samples. Did the authors use the imputed gene expression for the samples where expression data are missing? If true, this would lead to serious bias since the expression is confounded by so many factors (e.g. somatic mutations, copy number alterations, purity, etc) and thus unreliable through imputation by SNPs merely.

2.     The authors identified a strikingly higher proportion of trans-eQTLs in the AA sample (691 out of 706 eQTLs) than in the WW sample (266 out of 343 eQTLs). This is a very interesting (and might be important) finding indicating race difference in genetic architecture of cancer susceptibility. The authors might provide some explanations or thoughts on WHY AA sample shows a much higher proportion of trans-eQTLs than in WW sample, genetic structure/admixture? Is there evidence that normal tissues, e.g. in GTEx, also show similar patterns (higher trans-eQTLs than cis-eQTLs in AA versus WW)?

3.     Line 116 - "We attributed all but 7 of the cis-eGenes from CBCS across both AA and WW women found in GTEx to one of these three tissue types". The authors should describe what are these three tissue types in the text not just label them in Fig 1. What's more, the authors should explain why these three tissue types were considered. Of note, a substantial fraction of cis-eGenes are attributed to Adipose or Immune tissues rather Breast (Fig. 1b). All of the four common genes across race, PSPHL, GSTT2, EFHD1 and SLC16A3, are attributed to these two non-breast tissues. The author should clarify these findings.

4.     Line 124 - why is 417 genes not 406 genes here?  

5.     Line 125, "in WW women" in missing following "0.015 (𝑆𝐸 = 0.019)".

6.     Line 208 - The authors identified "4 whose GReX were associated with breast-cancer specific survival at FDR-adjusted 𝑃 < 0.10 in AA women" using the predicted expression data. Are these four genes also significant when only looking at samples with real expression data available?

7.     Supplemental Figure 2 legend- "Each point represents an eQTL with BBFDR<0.125 with the location of the 5' end of the corresponding eGenes on the X-axis and the genomic location of the corresponding eSNP on the Y-axis."" The description of X/Y axes should be switched.

8.     Discussion is lengthy (7 pages!) and should be shortened.

**Reviewer 2**

**Are you able to assess all statistics in the manuscript, including the appropriateness of statistical tests used?**

Yes, and I have assessed the statistics in my report.

**Comments to author:**

Overview:
Bhattacharya and colleagues present work on calling eQTLs in breast tumors measured in black and white women and impute gene expression to assess their impact on breast cancer survival. I found this study to be highly relevant due to the integration of cross-ancestry analyses in both the transcriptome level and survival outcomes. The manuscript is well-written and the results are presented clearly. That said I have several comments regarding the analysis of transcriptome data.

Major Comments:
1.     Is it possible to adjust for local CNVs in tumor expression? As the authors note, GSTT2 is a known CNV harboring gene. As a proof of principle can the authors impute CNVs using a tool (e.g, ensembleCNV) at GSTT2 and refit the eQTL model for both populations? This would give a good idea as to how susceptible these results are to CNV contamination.

2.     The authors presented a careful analysis of the eQTL mapping for women of African ancestry by including principal components to control for global ancestral differences (computed using both the Afr + Eur ancestry groups). However local ancestry differences are known to explain phenotypic variation in gene expression, and it would be extremely informative if the authors could condition on local ancestry for each cis-region tested as a secondary analysis.

3.     The authors performed genome-wide analysis for each of the 406 autosomal genes analyzed and identified 32 and 24 eGenes for the African and European ancestry groups respectively. Validation was performed by computing the correlation of estimated effect sizes with those from several GTEx tissues. While its reassuring that effect-sizes are similar, can the authors simply report if the lead eSNPs for a given eGene replicate in both GTEx and TCGA using p-values? This is critical for interpretation, most importantly for the trans-identified signals.

4.     While the main aim of the study is to leverage eQTLs to inform downstream brca survival risk, I think the manuscript would benefit from a functional analysis of the eQTL results. Using known functional annotations what are the enrichments for eQTLs across ancestry groups? This can be done in a straightforward manner using something like qtltools.

5.     Can the authors explain their use of LD-pruning before model fitting? The penalized linear models should be able to account for the LD structure naturally and still perform inference in ill-conditioned settings due to the penalty on the effect sizes. If the goal is to reduce the parameter space for computational reasons I would recommend filtering down to HapMap3 sites rather than LD pruning.

6.     The authors performed a stratified heritability analysis using GCTA, but it isn't clear what is the exact model used to estimate h2g. Is a the cish2g computed as the sum across the partitions for local while a trans component is conditioned on? The text would be much clearer if the authors could include the model used.

7.     I want to say that I really enjoyed seeing the TWAS BrCa risk analysis as a means to assess collider bias. Very thoughtful and reassuring that little evidence of bias was found.

Minor Comments:
1.     "AA" and "WW" abbreviations are used before they are defined on page 6. Same with "SRR" on Figure 1 legend and again with "EV" on page 10.
2.     Helpful to note that sample sizes per ancestry for eQTL analysis in results section (similar to SNPs , genes, etc). Is it the same numbers reported in the later predictive modeling section?
3.     Helpful to list h2g on table 1 alongside R2

**Authors Response**

**Point-by-point responses to the reviewers’ comments:**

*Response to reviewers for manuscript:

“A framework for transcriptome-wide association studies in breast cancer in diverse study populations”

We thank the reviewers for their helpful feedback which we believe has improved our manuscript. In summary, we have included results for follow-up analyses that span one additional supplemental table and 5 new supplemental figures. The major new analyses include: (1) functional enrichment of significant eQTLs identified in the Carolina Breast Cancer Study (CBCS), (2) additional analysis looking into the overlap of CBCS eQTLs with eQTLs reported in GTEx (across the three healthy tissues identified in the manuscript) and TCGA-BRCA, (3) a follow-up analysis of cis-eQTLs, conditioning on inferred local genetic ancestry, and (4) an analysis of the predictive performance of our expression models with and without linkage disequilibrium (LD) pruning of the genotype design matrix. These additional analyses contextualize our results further and reinforce our race-stratified eQTL analysis, construction of tumor expression models, and TWAS analysis.

We respond to each of the reviewers’ comments (prefaced in each case with ">") point by point below:*

> Reviewer 1
> Bhattacharya et al performed an integrative analysis of the eQTLs, gene expression predication and association with patient survival based on a large breast cancer cohorts CBCS with nearly equal representation of African American and White patients, and compared the results to TCGA. This integrative transcriptome-wide analysis (TWAS) allows the authors to uncover the dramatic difference between the two races in, e.g. the amount of trans versus cis-eQTLs, performance of gene expression prediction by germline variations, genes associated with patient survival, patterns in breast cancer subtypes, etc. This study underscores the importance of incorporating race or ancestry into TWAS analysis. Overall, this is an interesting and well-written manuscript. The authors should address the following comments in order to improve the quality of manuscript.

*We appreciate that the reviewer found our analysis important in addressing trans-ethnic genetic architectures in TWAS and the manuscript easy to follow.*

> 1. The authors should describe the sample size (AA and WW) in Results - Race specific germline eQTL analysis. According to Methods, the authors analyzed 5,989,134 germline SNPs from 3,828 samples (1,865 AA and 1,963 WW). However, the gene expression data (in 406 genes) are available only in 1,388 samples. It's not clear how the authors performed eQTL analysis with many of genotype-expression unpaired samples. Did the authors use the imputed gene expression for the samples where expression data are missing? If true, this would lead to serious bias since the expression is confounded by so many factors (e.g. somatic mutations, copy number alterations, purity, etc) and thus unreliable through imputation by SNPs merely.

*This is an important clarification point the reviewer makes, and we have cleared up the sample sizes for the eQTL analysis in Lines 96-98 of the revision: “We evaluated associations between the tumor expression levels of 406 autosomal genes and 5,989,134 germline SNPs in samples derived from 621 self-identified African American (AA) and 578 self-identified white women (WW).”

The eQTL analysis and predictive expression models were conducted and built only on samples that had all 406 autosomal genes assayed via Nanostring nCounter (621 AA samples and 578 WW samples). For transcriptomic imputation into CBCS and eventual TWAS association testing, we use the both genotype-expression paired and unpaired samples (3,828 total samples). We add more explicit details of the breakdown of the 3,828 total samples from CBCS in the Methods section (Lines 488-490).*

> 2. The authors identified a strikingly higher proportion of trans-eQTLs in the AA sample (691 out of 706 eQTLs) than in the WW sample (266 out of 343 eQTLs). This is a very interesting (and might be important) finding indicating race difference in genetic architecture of cancer susceptibility. The authors might provide some explanations or thoughts on WHY AA sample shows a much higher proportion of trans-eQTLs than in WW sample, genetic structure/admixture? Is there evidence that normal tissues, e.g. in GTEx, also show similar patterns (higher trans-eQTLs than cis-eQTLs in AA versus WW)?

*The reviewer’s question is very compelling. We first subjected our significant eQTLs to rigorous quality control by imposing a stricter minor allele frequency cutoff of 1% using the additive genotypes (i.e. 0, 1, or 2 minor alleles) and visual inspection. Details about this process are included in the Methods section. We have added Supplemental Figure 2 to show the plots we generated for visual inspection for eQTLs that failed this quality control. This resulted in the removal of 6 trans-eQTLs in the AA sample. Next, we have added some discussion of potential reasons why we find a greater proportion of trans-eQTLs in our AA sample (approximately 23% of total eQTLs as compared to approximately 5% in WW) in Lines 306-314:

“We also identified considerably more trans-eQTLs in the AA sample. This result may reinforce race differences in eQTL architecture as the ratio of detected trans-eQTLs to cis-eQTLs is not directly linked to sample size [40]. Differences in allele frequencies and linkage disequilibrium may contribute to observed differences in cis-eQTLs, as reported by Mogil et al [18], and we hypothesize that such differences may likewise affect trans-eQTLs, which were not analyzed by Mogil et al. Alternatively, there is a prevailing thought in literature about trans genetic regulation in admixed populations that the genetic diversity in individuals of African ancestry leads to added power of eQTL detection [41,42].

First, we acknowledge that, as we can see from the latest GTEx release (Figure 2C in [1]), sample size is not always correlated well with a larger number or ratio of trans-eQTLs. Furthermore, we hypothesize that differences in allele frequencies and linkage disequilibrium across ancestral groups could also contribute to differences in trans-eQTL architecture, based on the results Mogil et al show in cis-eQTLs [2]. Newly add Supplementary Figure 3 shows minor allele frequencies for all eSNPs across AA and WW samples. Lastly, we point to some ideas that both Brynedal et al and Shan et al mention, that genetic diversity of individuals of African ancestry may lead to increased power to detect trans-eQTLs [3,4].*

> 3. Line 116 - "We attributed all but 7 of the cis-eGenes from CBCS across both AA and WW women found in GTEx to one of these three tissue types". The authors should describe what are these three tissue types in the text not just label them in Fig 1. What's more, the authors should explain why these three tissue types were considered. Of note, a substantial fraction of cis-eGenes are attributed to Adipose or Immune tissues rather Breast (Fig. 1b). All of the four common genes across race, PSPHL, GSTT2, EFHD1 and SLC16A3, are attributed to these two non-breast tissues. The author should clarify these findings.

*We provide the names of these three tissues in the Results section (Lines 130-133):

“Similar to previous pan-cancer germline eQTL analyses [26], we cross-referenced eGenes found in CBCS with eGenes detected in relevant healthy tissues from Genotype-Tissue Expression (GTEx) Project: mammary tissue (breast), subcutaneous adipose, and EBV-transformed lymphocytes (immune) (see Methods)”

and give context and citations to their high relative composition in bulk breast tumor samples in Methods (Lines 549-551):

“We considered these tissues mainly due to their high relative composition in bulk breast tumor samples, as shown previously in many studies...”

Furthermore, we point out that, in GTEx version 7, the sample size for eQTL analysis in adipose tissue is significantly greater than those for lymphocytes and mammary tissue. Breast tumor tissue is a very heterogeneous tissue and, as Geeleher et al points out, an overwhelming majority of breast tumor eQTLs cannot be attributed to the tumor or the mammary tissue itself [5].*

> 4. Line 124 - why is 417 genes not 406 genes here?

*The reviewer was correct in pointing out this error. We only consider the 406 autosomal genes in the TWAS pipeline. Line 145 reflects this change.*

> 5. Line 125, "in WW women" in missing following "0.015 ( = 0.019)".

*We have corrected this oversight in Line 149.*

> 6. Line 208 - The authors identified "4 whose GReX were associated with breast-cancer specific survival at FDR-adjusted < 0.10 in AA women" using the predicted expression data. Are these four genes also significant when only looking at samples with real expression data available?

*This is an important question. We feel that we had already addressed in the manuscript. Please refer to Supplemental Figure 15, the last sentence of the Results section (Lines 290-292), and some discussion points in Lines 358-365:

“We did not observe any significant association between the total expression of these 4 genes and breast cancer-specific survival. This suggests that the germline-regulated component of the tumor expression of these genes – a small fraction of the total expression variation – may be associated with survival outcomes. Numerous factors, including copy number alterations, epigenetic or post-transcriptional regulation, and exposures and technical artifacts in measurement contributed to the total expression measured in the tumor. Thus, we do not expect that significant GReX association implies total expression association, or vice versa.”*

> 7. Supplemental Figure 2 legend- "Each point represents an eQTL with BBFDR<0.125 with the location of the 5' end of the corresponding eGenes on the X-axis and the genomic location of the corresponding eSNP on the Y-axis."" The description of X/Y axes should be switched.

*We have corrected the caption for this supplemental figure. We thank the reviewer for catching this error.*

> 8. Discussion is lengthy (7 pages!) and should be shortened.

*We agree that the Discussion section was previously too long and have tightened the language and removed redundant restatements of the results, beyond the first paragraph summary. The Discussion paragraphs now cover a summary, race-specific eQTL architecture, race-specific predictive expression models, literature on TWAS-detected loci, mention of the collider bias literature, and limitations of the study.*

-----

> Reviewer 2
> Bhattacharya and colleagues present work on calling eQTLs in breast tumors measured in black and white women and impute gene expression to assess their impact on breast cancer survival. I found this study to be highly relevant due to the integration of cross-ancestry analyses in both the transcriptome level and survival outcomes. The manuscript is well-written and the results are presented clearly. That said I have several comments regarding the analysis of transcriptome data.

*We appreciate that the reviewer found our analysis relevant for the current literature of cross-ancestry analyses and the manuscript well-written and clear.*

> Major Comments:
> 1. Is it possible to adjust for local CNVs in tumor expression? As the authors note, GSTT2 is a known CNV harboring gene. As a proof of principle can the authors impute CNVs using a tool (e.g, ensembleCNV) at GSTT2 and refit the eQTL model for both populations? This would give a good idea as to how susceptible these results are to CNV contamination.

*Unfortunately, somatic mutation data is not available in the CBCS and thus somatic copy number variation cannot be imputed. We point out this limitation of the CBCS data in the final paragraph of the Discussion in the context of the predictive expression models:

“One limitation of our study is that data on somatic amplifications and deletions were not yet available for the CBCS cohort we analyzed. Removing the somatic copy number variation signal from tumor expression profiles may improve our estimates of cis-heritability and perhaps the predictive performance of our models, though previous TWAS in ovarian cancer shows the effect to be qualitatively small (approximately less than 2% change in heritability) [47].”

We point out that the gain in predictive power may only be marginal, as Gusev et al shows in their TWAS analysis in ovarian cancer [6].*

> 2. The authors presented a careful analysis of the eQTL mapping for women of African ancestry by including principal components to control for global ancestral differences (computed using both the Afr + Eur ancestry groups). However local ancestry differences are known to explain phenotypic variation in gene expression, and it would be extremely informative if the authors could condition on local ancestry for each cis-region tested as a secondary analysis.

*We thank the reviewer for this suggestion, which is one of the significant additions to our manuscript. We followed the pipeline outlined by Martin et al 2017 to infer the estimated haplotype dosage of African ancestry at each SNP site using RFMix [7,8]. We then use the local ancestry-adjusted eQTL framework proposed by Zhong et al 2019 to follow up on our cis-eQTL analysis, conditioning of local ancestry estimates [9]. In this local ancestry-adjusted eQTL analysis, we consider only the genome-wide ancestry-adjusted cis-eGenes that we detected in only one of the AA or WW samples. We discuss all details in the Methods section.

In summary, with local ancestry adjustment, we show, generally, marginal increases in the strength of the association between lead cis-eSNP and eGene, as shown by the distribution of the difference in -values between local ancestry-adjusted and genome-wide ancestry-adjusted eQTL associations in Supplemental Figure 7A (with a mode of 0.5 on the -log10 scale, meaning eQTL P-values tended to be lower after local ancestry adjustment by a factor of ~3). However, local ancestry adjustment does not fully harmonize eQTL association across race, as seen in Supplemental Figure 7B and 7C where we show the difference in –log10 P-value across race before and after local ancestry adjustment. We discuss these results and some complications in relying on two source populations (CEU and YRI source populations in the 1000 Genomes Project) in detail in Supplemental Results.

We feel that a more extensive analysis of the impact of local ancestry adjustment into both the eQTL analysis and predictive tumor expression models will be compelling as a future extension.*

> 3. The authors performed genome-wide analysis for each of the 406 autosomal genes analyzed and identified 32 and 24 eGenes for the African and European ancestry groups respectively. Validation was performed by computing the correlation of estimated effect sizes with those from several GTEx tissues. While its reassuring that effect-sizes are similar, can the authors simply report if the lead eSNPs for a given eGene replicate in both GTEx and TCGA using p-values? This is critical for interpretation, most importantly for the trans-identified signals.

*We have added Supplemental Figure 9 that shows the correlation of -values of eQTLs that we found in GTEx adipose tissue, mammary tissue, and lymphocytes, as well as in TCGA-BRCA using the PancanQTL database [10]. We discuss these results shortly in Lines 137-143.

We were unable to replicate the CBCS trans-eQTLs in the GTEx tissues we prioritized and TCGA-BRCA. We make a few points about potential reasons why we think CBCS trans-eQTLs were not identified in GTEx and TCGA-BRCA: (1) The majority of trans-eGenes were identified in AA women. GTEx and TCGA-BRCA do not have large African American sample sizes. (2) We also discuss the higher number of trans-eQTLs in AA women compared to WW women in the Discussion section, as Reviewer 1 requested.*

> 4. While the main aim of the study is to leverage eQTLs to inform downstream brca survival risk, I think the manuscript would benefit from a functional analysis of the eQTL results. Using known functional annotations what are the enrichments for eQTLs across ancestry groups? This can be done in a straightforward manner using something like qtltools.

*We have added a functional enrichment analysis using QTLtools [11], assessing the number of eQTLs identified in CBCS that fall within 1 kilobase of DNase1 hypersensitive sites in the MCF-7 breast cancer cell line and transcription factor binding sites in the T-47D breast cancer cell line. We added details on this analysis in Methods. We find that eQTLs identified in WW women show strong enrichment in these DNase1 cleavage hotspots (~20 fold enrichment, P-value=) and transcription factor binding sites (7.5 fold enrichment, P-value=0.002), but no such enrichment in the AA eQTLs. We add a new Supplemental Table 1 which summarizes these results and point to the table in Lines 111-117.*

> 5. Can the authors explain their use of LD-pruning before model fitting? The penalized linear models should be able to account for the LD structure naturally and still perform inference in ill-conditioned settings due to the penalty on the effect sizes. If the goal is to reduce the parameter space for computational reasons I would recommend filtering down to HapMap3 sites rather than LD pruning.

*Chatterjee et al 2016 mentions that LD pruning has been historically used for feature selection to aid in training polygenic scores, warning nevertheless against overly stringent pruning [12]. We believe that our cutoff of 0.5 and liberal window sizes does not eliminate independent association signals. We have added this citation to the manuscript.

During the research process, we originally did not prune for LD in the design matrix, but we saw increased predictive performance in cross-validation after LD pruning. For all genes with significant cis-heritability, we trained expression models without LD pruning and compared the cross-validated in a newly added Supplemental Figure 18. Models that were trained with pruned design matrices generally showed greater predictive performance.*

> 6. The authors performed a stratified heritability analysis using GCTA, but it isn't clear what is the exact model used to estimate h2g. Is a the cish2g computed as the sum across the partitions for local while a trans component is conditioned on? The text would be much clearer if the authors could include the model used.

*We have provided more details about the scheme (GREML-LDMS [13]) used to estimate the cis-heritability (cis- of tumor gene expression in Methods:

“Briefly, Yang et al shows that estimates of heritability are often biased if causal variants have a different minor allele frequency (MAF) spectrums or LD structures from variants used in analysis. They proposed an LD and MAF-stratified GREML analysis, where variants are stratified into groups by MAF and LD, and genetic relationship matrices (GRMs) from these variants in each group are jointly fit in a multi-component GREML analysis. Extensive details are given by Yang et al [28].”

We also added that we do not consider the trans component in this heritability estimation.*

> 7. I want to say that I really enjoyed seeing the TWAS BrCa risk analysis as a means to assess collider bias. Very thoughtful and reassuring that little evidence of bias was found.

*We thank the reviewer for appreciating this assessment of collider bias.*

> Minor Comments:
> 1. "AA" and "WW" abbreviations are used before they are defined on page 6. Same with "SRR" on Figure 1 legend and again with "EV" on page 10.
> 2. Helpful to note that sample sizes per ancestry for eQTL analysis in results section (similar to SNPs, genes, etc). Is it the same numbers reported in the later predictive modeling section?
> 3. Helpful to list h2g on table 1 alongside R2

*We thank the reviewer for these minor comments that improve the readability of the manuscript and have accordingly addressed them.

-----

References

1. Aguet F, Barbeira AN, Bonazzola R, Brown A, Castel SE, Jo B, et al. The GTEx Consortium atlas of genetic regulatory effects across human tissues. bioRxiv [Internet]. Cold Spring Harbor Laboratory; 2019 [cited 2019 Nov 15];787903. Available from: https://www.biorxiv.org/content/10.1101/787903v1
2. Mogil LS, Andaleon A, Badalamenti A, Dickinson SP, Guo X, Rotter JI, et al. Genetic architecture of gene expression traits across diverse populations. Epstein MP, editor. PLOS Genet [Internet]. Public Library of Science; 2018 [cited 2019 Jun 13];14:e1007586. Available from: https://dx.plos.org/10.1371/journal.pgen.1007586
3. Brynedal B, Choi JM, Raj T, Bjornson R, Stranger BE, Neale BM, et al. Large-Scale trans-eQTLs Affect Hundreds of Transcripts and Mediate Patterns of Transcriptional Co-regulation. Am J Hum Genet. Cell Press; 2017;100:581–91.
4. Shan N, Wang Z, Hou L. Identification of trans-eQTLs using mediation analysis with multiple mediators. BMC Bioinformatics. BioMed Central Ltd.; 2019;20.
5. Geeleher P, Nath A, Wang F, Zhang Z, Barbeira AN, Fessler J, et al. Cancer expression quantitative trait loci (eQTLs) can be determined from heterogeneous tumor gene expression data by modeling variation in tumor purity. Genome Biol [Internet]. BioMed Central; 2018 [cited 2019 Apr 30];19:130. Available from: https://genomebiology.biomedcentral.com/articles/10.1186/s13059-018-1507-0
6. Gusev A, Lawrenson K, Lin X, Lyra PC, Kar S, Vavra KC, et al. A transcriptome-wide association study of high-grade serous epithelial ovarian cancer identifies new susceptibility genes and splice variants. Nat Genet [Internet]. Nature Publishing Group; 2019 [cited 2019 Jun 5];51:815–23. Available from: http://www.nature.com/articles/s41588-019-0395-x
7. Martin AR, Gignoux CR, Walters RK, Wojcik GL, Neale BM, Gravel S, et al. Human Demographic History Impacts Genetic Risk Prediction across Diverse Populations. Am J Hum Genet. Cell Press; 2017;100:635–49.
8. Maples BK, Gravel S, Kenny EE, Bustamante CD. RFMix: A discriminative modeling approach for rapid and robust local-ancestry inference. Am J Hum Genet. 2013;93:278–88.
9. Zhong Y, Perera MA, Gamazon ER. On Using Local Ancestry to Characterize the Genetic Architecture of Human Traits: Genetic Regulation of Gene Expression in Multiethnic or Admixed Populations. Am J Hum Genet. Cell Press; 2019;104:1097–115.
10. Gong J, Mei S, Liu C, Xiang Y, Ye Y, Zhang Z, et al. PancanQTL: Systematic identification of cis -eQTLs and trans -eQTLs in 33 cancer types. Nucleic Acids Res. Oxford University Press; 2018;46:D971–6.
11. Delaneau O, Ongen H, Brown AA, Fort A, Panousis NI, Dermitzakis ET. A complete tool set for molecular QTL discovery and analysis. Nat Commun. Nature Publishing Group; 2017;8.
12. Chatterjee N, Shi J, García-Closas M. Developing and evaluating polygenic risk prediction models for stratified disease prevention. Nat. Rev. Genet. Nature Publishing Group; 2016. p. 392–406.
13. Yang J, Bakshi A, Zhu Z, Hemani G, Vinkhuyzen AAE, Lee SH, et al. Genetic variance estimation with imputed variants finds negligible missing heritability for human height and body mass index. Nat Genet [Internet]. NIH Public Access; 2015 [cited 2019 May 29];47:1114–20. Available from: http://www.ncbi.nlm.nih.gov/pubmed/26323059*

**Second round of review**

**Reviewer 1**

My questions and concerns have been well addressed in the revised manuscript.

**Reviewer 2**

The authors have addressed all my comments in a satisfactory way. I commend them on their efforts undertaking the number of new analyses that help place results in a broader context.
